# Barriers to and facilitators of maintaining physical activity for people with hip and knee osteoarthritis: a mixed-methods systematic review

**DOI:** 10.1007/s00296-026-06230-0

**Published:** 2026-07-01

**Authors:** Peter Hempenstall, Grainne Hayes, Catherine Woods, Suzanne McDonough, Aoife Stephenson, Clodagh M. Toomey

**Affiliations:** 1 School of Allied Health, University of Limerick, Limerick, Ireland; 2 Department of Physical Education and Exercise Sciences, University of Limerick, Limerick, Ireland; 3 School of Physiotherapy, RCSI University of Medicine and Health Sciences, Dublin, Ireland; 4 Health Research Institute, University of Limerick, Limerick, Ireland; 5 Physical Activity for Health Research Centre, University of Limerick, Limerick, Ireland; 6 Department of Sport Science and Clinical Biomechanics, Research Unit for Musculoskeletal Function and Physiotherapy, University of Southern Denmark, Odense, Denmark

**Keywords:** Osteoarthritis, Physical activity, Systematic Review, Maintenance, Barrier, Facilitator

## Abstract

Increasing physical activity (PA) is an important recommendation for the management of hip and knee osteoarthritis (OA), under which components of therapeutic exercise can be embedded. However, maintaining PA behaviour (≥ 6-months) following the cessation of structured exercise interventions remains challenging and under-explored. This mixed-methods systematic literature review aimed to identify the barriers to and facilitators of maintaining PA behaviour for those living with hip and/or knee OA. Electronic databases were searched for peer-reviewed studies examining PA behaviour maintenance (defined as at least six-months post-intervention) for those with hip and/or knee OA. Data were extracted, appraised using the Mixed-methods Appraisal Tool (MMAT), and synthesised narratively. Sixteen studies (*n* = 1,486; 73.7% female) were included: six quantitative, five qualitative and five mixed-methods. The most frequently cited TDF domains were *beliefs about capabilities* (e.g. confidence reinforced by intervention skills), *environmental context and resources* (e.g. competing priorities), and *memory*,* attention and decisional processes* (e.g. memory recall - forgetting). Quantitative evidence supported these domains highlighting that correlates such as self-efficacy, energy levels, social support and resource availability strongly influenced PA behaviour maintenance. Integrated findings informed the Physical Activity Maintenance for osteoArthritis (PAMA) conceptual map, illustrating the dynamic interactions between individual, social and environmental factors. This review revealed that maintaining PA behaviour is multifactorial and influenced by individual, social and environmental factors. Further research should consider these interactions in the design of future interventions and in healthcare practice to foster and support the maintenance of PA behaviour for those living with hip and knee OA. PROSPERO registration: CRD42024591820.

**Supplementary Information:**

The online version contains supplementary material available at 10.1007/s00296-026-06230-0.

## Introduction

Osteoarthritis (OA) is a painful and debilitating degenerative disease characterised by the deterioration of articular cartilage and associated structural and inflammatory changes in the tissues surrounding the joint, including subchondral bone, muscles, and ligaments [1, 2]. It is a leading cause of pain, reduced mobility and loss of functional independence among older adults, significantly impacting their quality of life (QOL) [2–4]. It has become one of the fastest growing health problems in the world, affecting over 595 million people worldwide [5]. Unfortunately, no cure exists for OA; however, various treatment strategies aim to minimise pain, symptoms and improve function. As a result, it is essential to identify and implement effective, sustainable strategies that will support the long-term management of OA, helping individuals maintain mobility, alleviate symptoms and improve QOL.

Physical activity (PA), defined as any bodily movement that results in energy expenditure encompasses both structured and unstructured forms of movement [6]. Exercise is a subcategory of PA that is planned, structured, repeated and aimed at improving or maintaining components of physical fitness and health [7, 8]. Therapeutic exercise in contrast, refers to exercises designed to address specific impairments associated with a condition such as OA [8]. In the context of OA management, therapeutic exercise is commonly delivered by trained healthcare professionals through structured programmes such as GLA: D^®^ (Good living with osteoarthritis from Denmark) and the ESCAPE-Knee pain (Enabling Self-management and Coping with Arthritic knee Pain through Exercise) programme. These types of interventions are widely recommended as first-line treatments by major international clinical guidelines [9–11] and have consistently demonstrated short-term improvements in pain, physical function and QOL for those living with hip and knee OA [12–15].

However, despite initial positive outcomes, symptoms often return to baseline and benefits attained begin to diminish following reduced PA participation [16–18]. A recent review identified that therapeutic exercise programmes are often designed with a focus on short-term benefits, rather than supporting the long-term management of OA [17]. Since 2019, EULAR (European Alliance of Associations for Rheumatism) therefore suggests that therapeutic exercise should not be viewed as the remedy for OA management but rather as a bridge towards sustaining long-term PA participation in daily life [8]. In 2023, EULAR re-emphasised this highlighting that exercise should be embedded within a broader PA plan, inclusive of both aerobic PA and muscle-strengthening activities [10]. Within this, long-term PA behaviour maintenance refers to continued participation in PA at levels sufficient to meet public health guidelines, rather than ongoing adherence to structured therapeutic exercise alone.

This broader conceptualisation of PA includes both structured activities (e.g. supervised exercise programmes) and unstructured or lifestyle-based activities (e.g. walking and other forms of daily movement). Evidence indicates that such activities when supported by behavioural strategies such as goal-setting can improve pain and function in individuals with OA and other musculoskeletal conditions [19–21]. Importantly, these interventions may enable the transition from structured, OA-specific exercise to more autonomous, sustainable forms of PA behaviour.

Despite this knowledge, a significant number of people living with hip and/or knee OA still struggle to maintain PA behaviour with approximately one in three individuals reporting a decline in PA at 12-month follow-up [12]. Broader behaviour change models such as the Transtheoretical Model (TTM), indicate that this decline at 12-months suggests difficulties in sustaining PA beyond the recognised threshold for maintenance, defined as a behaviour performed consistently for six or more months [22, 23]. These findings therefore highlight an evidential gap to understand what supports or hinders PA behaviour maintenance as those living with hip and knee OA transition from structured exercise to unstructured sustained PA.

While barriers and facilitators to PA for hip and knee OA have been investigated with respect to adoption and adherence in various reviews [24–26], far less documented are the factors influencing the maintenance of PA behaviour once structured exercise programmes conclude. Despite some qualitative studies exploring these determinants in isolation [27–29], no comprehensive review has brought together and synthesised both quantitative and qualitative evidence for the long-term maintenance of PA behaviour for hip and knee OA. Addressing this gap is instrumental for informing future behavioural interventions to maximise clinical effectiveness for supporting long-term PA – a behaviour repeatedly linked with improved outcomes and reduced healthcare burden in those living with OA [30, 31].

Therefore, this review aimed to address this critical gap by synthesising the existing literature on barriers and facilitators to maintaining PA post-programme in those living with hip and knee OA. Through the integration of qualitative and quantitative evidence, this study provides a comprehensive understanding of the individual, social and environmental factors that shape long-term PA behaviour. These findings may guide interventions in supporting the transition from structured exercise to sustained long-term PA behaviour for those living with hip and knee OA.

## Methods

This systematic literature review hereafter: referred to as systematic review was registered with the International prospective register of systematic reviews (PROSPERO) with the registration number: CRD42024591820. The protocol for this study has also published previously [22]. The reporting of this systematic review is in accordance with the Preferred Reporting Items for Systematic Reviews and Meta-Analysis (PRISMA) checklist.

### Search strategy

A search was developed in consultation with a research librarian from the University of Limerick and conducted using a comprehensive list of Boolean search terms on the following databases: APA PsycINFO, CINAHL Complete, Cochrane library, Embase, Medline via PubMed and the Web of Science. This search was first conducted on Medline via PubMed and then modified and transposed to each individual database. This search was initially conducted on the 28th of February 2025 and again on the 25th of March 2026 using the same search strategy to account for recent publications. Further details of this search are provided in the supplementary material.

### Eligibility criteria

The eligibility criteria has been discussed elsewhere [22] however; briefly, studies were included if they:

- Were peer-reviewed original research articles reported in English.
- Included adults with hip and/or knee OA.
- Involved participants who had previously completed a PA intervention at least six-months before outcome assessment (e.g. structured exercise programmes, multicomponent exercise programmes (e.g. diet and exercise) or provided with self-management strategies).
- Investigated barriers and/or facilitators to maintaining PA behaviour (defined as ≥ 6-months). Maintaining PA behaviour could include continuing with daily exercises or being more physically active in their daily life; and.
- Employed qualitative, quantitative, or mixed-methods designs, including observational designs (e.g. cross-sectional, longitudinal, or cohort studies) examining PA behaviour maintenance.

Studies were excluded if they were book chapters, review articles, theoretical or opinion papers, or did not meet the inclusion criteria outlined above.

### Study selection

All retrieved records were imported to Covidence and duplicates removed. A rapid screen of studies was performed whereby clearly irrelevant studies such as those on non-human participants and systematic reviews were removed (*n* = 1473). Title and abstracts were then screened by two independent reviewers (PH, CT) using the pre-defined eligibility criteria. Conflicts were resolved following discussion among authors until consensus reached. All eligible full-texts were reviewed by PH and independently by four co-authors. Conflicts were resolved through discussion and an independent verifier where required.

### Data extraction and quality assessment

For included studies, data were extracted by the lead author (PH) and verified by an independent reviewer (CT). Data extracted from each article was imported and managed using Microsoft Excel (version 2602) and included: author, year, title, country, study characteristics (study design, study methods and analysis), participant characteristics (population of interest, sample size, affected joint, additional comorbidities), intervention characteristics (intervention, length of follow-up, additional behaviour change techniques used) and findings (key findings, barriers and facilitators). For qualitative studies and qualitative components of mixed-methods studies, all quotations on barriers and facilitators to maintaining PA behaviour were also extracted. The included articles were assessed for methodological quality using the Mixed Methods Appraisal Tool (MMAT-version 2018) [32]. One independent reviewer (PH) conducted a quality assessment on all included studies (*n* = 16), and two independent reviewers (CT, GH) reviewed 8 papers each. Studies were rated as high (meeting ≥ 75% of quality criteria), medium (50–74% of criteria), low (25–49% of criteria) or critically low-quality (≤ 24% of criteria). This was used to inform the data synthesis and significance of the findings of the included studies. Details of this can be seen in Table 1 and further in supplementary material.

### Synthesis of results

Given the heterogeneity of the study designs and outcome measures, a narrative synthesis as proposed by Yang et al. [33] was used to analyse the data. First, the qualitative studies and qualitative components of mixed-methods studies were analysed using a thematic analysis [34] and followed the steps set out in the original protocol [22, 35]. Briefly, this followed the thematic analysis process through (1) data-set familiarisation, followed by (2) line-by-line coding to generate initial codes, (3) grouping of codes and searching for themes, (4) development of descriptive themes, and (5) through an iterative process of review and revision, refinement of the descriptive themes into analytical themes. This was conducted by the lead author (PH) and verified by a second author (CT). These themes were subsequently mapped to the theoretical domains framework (TDF) [36], providing an overarching framework and a theoretical basis for understanding the determinants of behaviour change. The barriers and facilitators to maintaining PA behaviour reported in the quantitative studies and quantitative components of mixed-methods studies were tallied and subsequently, were also mapped to the TDF to enable the integration of evidence across study types. As recognised by the authors in designing the TDF, specific factors may occur across various domains, rendering that these domains do not remain mutually exclusive [37]. Therefore, mapping each individual theme to the TDF domains and constructs was performed through an iterative process of continual revision and refinement. This was performed by the lead author and verified by a second author (CT). Where discrepancies arose, consensus was reached following discussion among authors. For further information, including descriptions underpinning translation of themes to TDF domains, please see supplementary material.

### Development of the physical activity maintenance for osteoarthritis (PAMA) conceptual map

Given the diverse evidence identified in this review, a conceptual map was developed to illustrate the key factors influencing PA behaviour maintenance. This approach as proposed by Novak and Cañas [38] supports the organisation and representation of knowledge in relation to a defined focus question. Following this approach, the process involved defining the focus question (identifying barriers and facilitators to maintaining PA behaviour), identifying and listing the key concepts, constructing a preliminary map and iteratively refining it through continuous review and revision. In line with the integrative nature of the TDF, which encompasses individual, social and environmental determinants of behaviour [36], findings from this review were organised into three overarching categories: individual, social and environmental factors. While the mapping process followed the general steps outlined by Novak and Cañas [38], it did not incorporate hierarchical structuring or cross-link development. Instead, it was informed by the approach described by Artinian [39], which emphasises understanding the processes through which outcomes occur, rather than exploring the amount of variance between factors. Further details of the conceptual mapping process are provided below in the supplementary material.

**Fig. 1:**
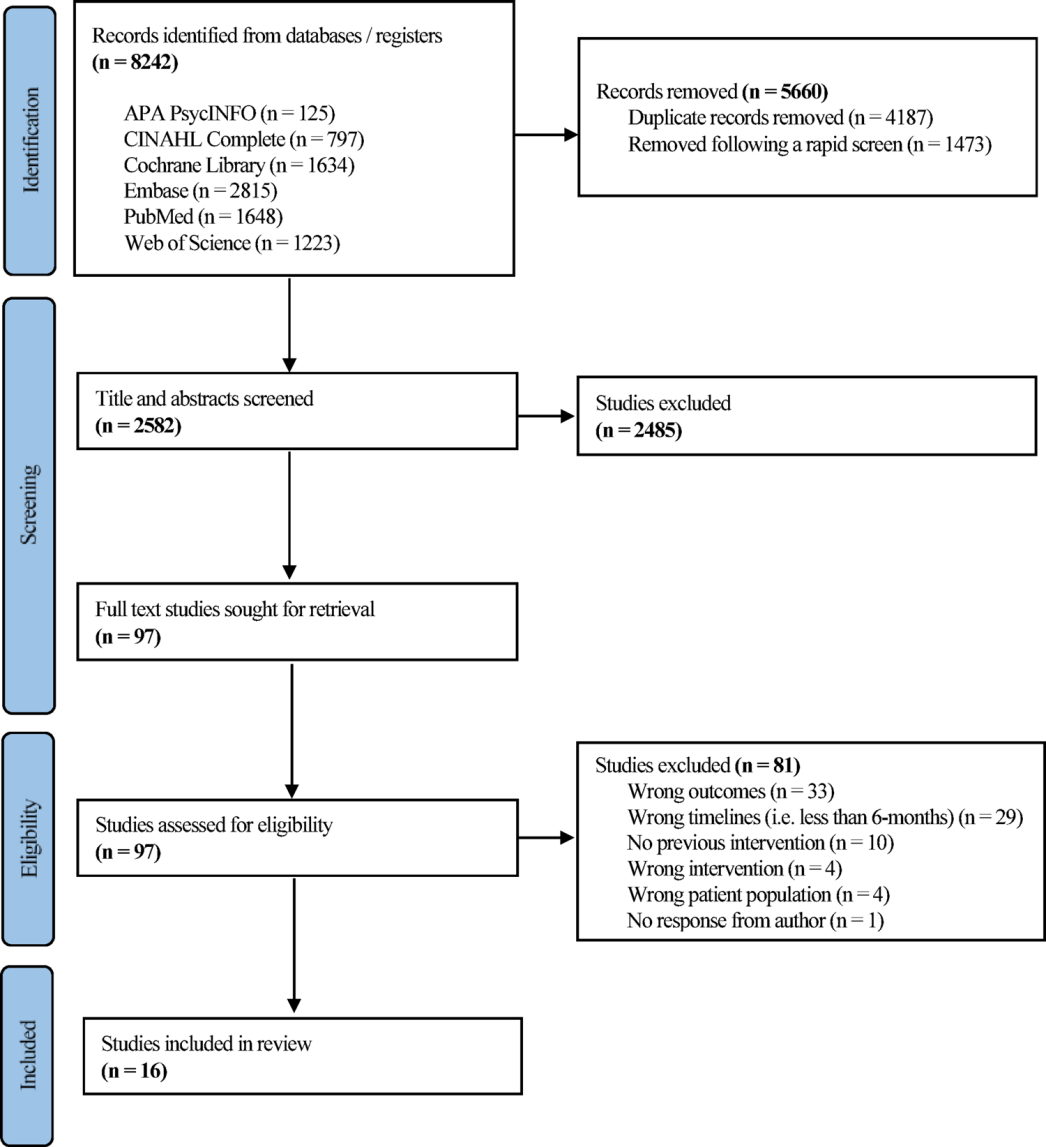
PRISMA Flowchart

## Results

### Study selection

Following the systematic search, 8242 studies were identified. After duplicate removal and following a rapid screen, 2582 articles were imported to Covidence and underwent title and abstract screening with 97 studies eligible for full-text review. Two authors were contacted for further information, of which one provided information. A total of 16 studies met eligibility and were included for data extraction. This process has been outlined in the PRISMA flow chart (Fig. 1).

### Study characteristics

Characteristics of the 16 included studies are summarised in Table 1. In total, 1,486 participants were represented across studies, with females comprising 73.7% of the overall sample. Most study samples focused on individuals with knee OA (*n* = 11) [27, 40–49], while fewer investigated hip OA (*n* = 2) [50, 51] or both hip and knee OA (*n* = 3) [52–54]. Of the included studies, six employed quantitative methods [40, 43, 45, 48, 50, 52], five used qualitative approaches [27, 44, 46, 47, 54], and five utilised a mixed-methods design [41, 42, 49, 51, 53]. Five studies were conducted in Australia, five in the USA, two in the UK, and one each in Denmark, Netherlands, Singapore and Switzerland. Study follow-ups ranged from 6-months to 6 years with 14 studies investigating barriers and facilitators within a 12-month window (range 6- to 18-months).

### Risk of bias

Table 1 details the quality assessment of the included studies with 19% of studies (*n* = 3) found to be of medium quality and 81% (*n* = 13) of the included studies considered high quality. Agreement was high across reviewers with agreement rates of 91.21% and 92.42% between independent reviewer one (PH) with independent reviewer 2 (CT), and 3 (GH), respectively. For further information on quality assessment criteria, please see supplementary material.

**Fig. 2:**
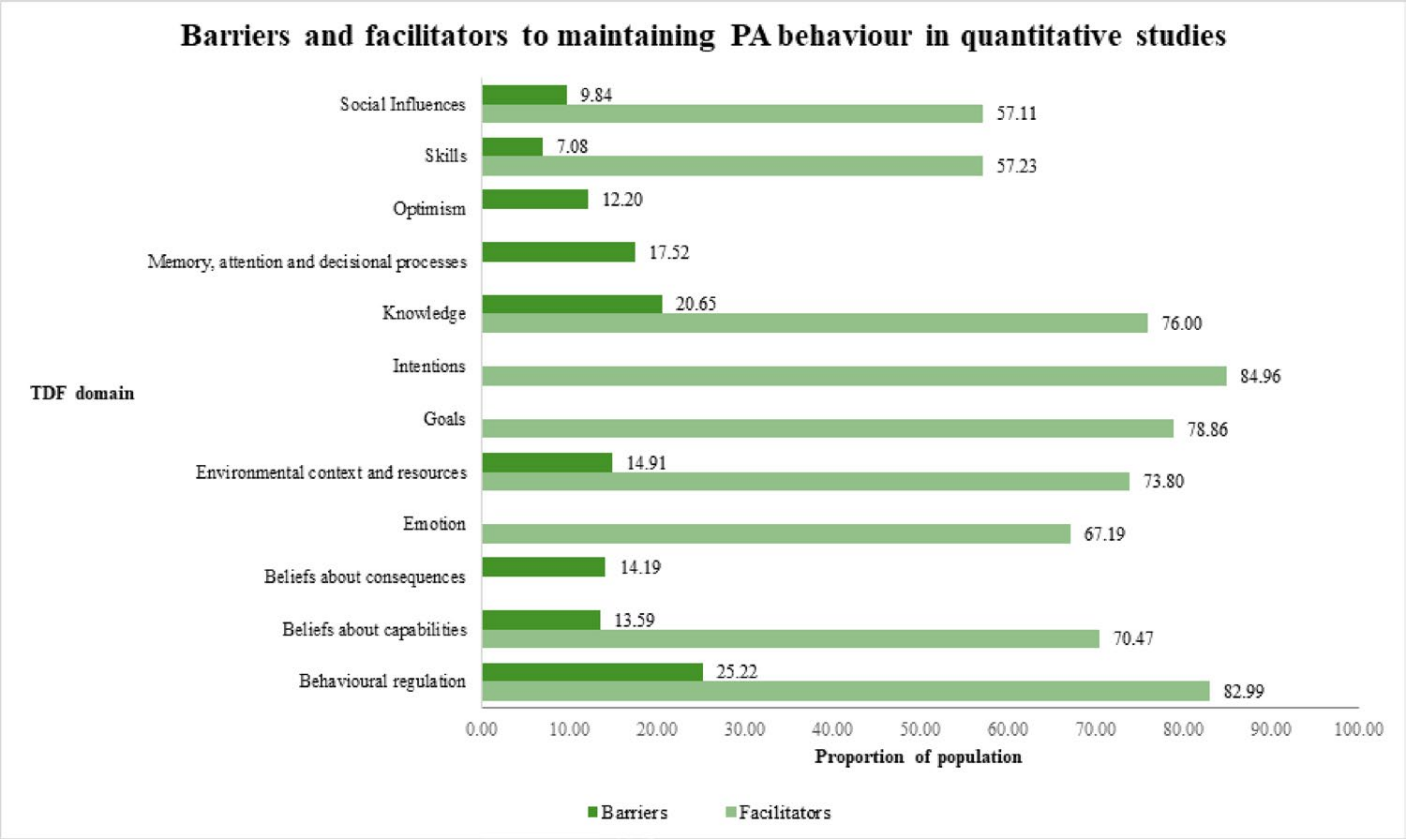
Barriers and facilitators to maintaining physical activity (PA) behaviour in quantitative studies and quantitative components of mixed-methods studies, by mean proportion of participants across the theoretical domains framework (TDF) domains

**Table 1:** Characteristics of the included studies

Author, year	Country	Study design	Population characteristics	Duration for follow-up	MMAT quality
Bennell et al., 2020 [40]	Australia	Quantitative, a parallel two-group superiority RCT	*N* = 110 participants (67.2% female) Mean age: SMS group (61.7 ± 6.7 years), Control group 62.9 ± 6.8 years), knee OA	6-months	High (100%), high quality on all criteria.
Cheung et al., 2022 [42]	USA	Mixed-methods, Qualitative: Focus group interviews, quantitative: exercise diaries	*N* = 28 participants (100% female), Mean age: 71.2 ± 7.8 years, knee OA	6- and 12-months	High (82.4%), lacked clarity on discussing divergences between qualitative and quantitative findings.
Cheung et al., 2015 [41]	USA	Mixed-methods, cross-sectional descriptive design using survey, interviews and video analysis	*N* = 31 participants (100% female), Mean age: 72 ± 5.6 years, knee OA	6-months	Medium (73.5%), lacked clarity on rationale to; and integration of using a mixed-methods design to answer the research question.
Desai et al., 2014 [52]	USA	Quantitative, mixed-effects modelling of a randomised PA trial	*N* = 486 participants (86.6% female), Mean age: 71.1 years, range (59 to 91 years), hip /knee OA	6-, 12-, and 18-months	High (78.6%), partial non-complete outcome data, unclear if participants representative of target population.
Hammer et al., 2015 [51]	Denmark	Mixed-methods, a parallel mixed data analysis design	*N* = 52 participants (69% female), Mean age: 69 years, range (65–74 years), hip OA	8-months	High (91.2%), co-founders not accounted for in data analysis
Hinman et al., 2023 [44]	Australia	Qualitative, semi-structured interviews	*N* = 26 participants (68% female), Mean age: responders (57 ± 7 years), non-responders (67 ± 9 years), knee OA	6-months	High (100%), high quality on all criteria.
Hinman et al., 2020 [43]	Australia	Quantitative, a pragmatic superiority parallel-group RCT	*N* = 175 participants (62.86% female), Mean age: Existing service (62.5 ± 8.1 years), Exercise advice and support group (62.4 ± 9.1 years), knee OA	6-months	High (100%), high quality on all criteria.
Kawi et al., 2015 [45]	USA	Quantitative, a two arm quasi-experimental pilot study	*N* = 16 participants (100% female), Mean age: 60.9 years range (52–72 years), knee OA	6-months	High (92.9%), unclear if co-founders accounted for in data analysis
Lawford et al., 2023 [46]	Australia	Qualitative, semi-structured interviews	*N* = 20 participants (50% female), Mean age: 65 ± 9 years, knee OA	6-months	High (100%), high quality on all criteria.
Ledingham et al., 2019 [47]	USA	Qualitative, semi-structured interviews	*N* = 25 participants (84% female), Mean age: 67 ± 6.1 years, knee OA	24-months	High (100%), high quality on all criteria.
Matil et al., 2024 [53]	Switzerland	Mixed-methods, exploratory sequential design	*N* = 350 participants (66.6% female), Mean age: Quantitative (67 ± 9.3 years), Qualitative (64 ± 9.3 years), hip/knee OA	5–17 months	High (88.2%), qualitative interpretation; and coherence between data sources, collection, analysis and interpretation was unclear
Moore et al., 2020 [27]	UK	Qualitative, longitudinal, semi-structured interviews	*N* = 30 participants (50% female), Mean age: Not specified however age for inclusion: (≥ 45 years), knee OA	12-months	High (100%), high quality on all criteria.
Stanton et al., 2020 [48]	Australia	Quantitative, feasibility study using RCT	*N* = 20 participants (70% female), Mean age: 67 ± 7.4 years, knee OA	6-months	Medium (71.4%), groups differed significantly at baseline on WOMAC; partial incomplete outcome data.
Tan et al., 2025 [49]	Singapore	Mixed-methods, explanatory sequential design	*N* = 22 participants (63.6% female), Mean age: 68.3 ± 6.92 Years, knee OA	6-months	High (94.12%), unclear of quantitative data reported in full.
Veenhof et al., 2006 [54]	Netherlands	Qualitative, open-ended in-depth 1:1 interviews	*N* = 12 participants (74% female), Mean age: 70 years (range 55–80 years), hip/knee OA	15–20 months	High (100%), high quality on all criteria.
Wainwright et al., 2020 [50]	UK	Quantitative, cross-sectional survey	*N* = 83 participants (54% female), Mean age: 67.35 ± 8.59 years, hip OA	4–6 years	Medium (71.4%), measurements lacked clarity to appropriately addressing research question.

*OA,* Osteoarthritis, *RCT* Randomised Control Trial, *SMS* Short message service, *UK* United Kingdom, *USA* United States of America, *WOMAC* Western Ontario and McMaster Universities Osteoarthritis Index

## Results of synthesis

Findings from all 16 included studies were mapped to the TDF. The findings from the quantitative designs and quantitative components of mixed-methods designs (*n* = 11) revealed barriers and facilitators to maintaining PA behaviour were present in 12 of the 14 TDF domains as represented in Fig. 2. Across all 16 included studies; the five most frequently cited domains were *beliefs about capabilities*, *environmental context and resources*, *memory*,* attention and decisional processes*,* social influences* and *beliefs about consequences*, respectively. These collective TDF domains captured interrelated influences on motivation, capability and opportunity underpinning the maintenance of PA behaviour. Although measurement of PA behaviour varied across studies from objective monitoring to self-report, the following results have been discussed collectively. The number of studies reporting each domain and exemplar quotes for each domain is shown in Table 2. However, for full inductive thematic analysis, mapping to the TDF domains, exemplar quotes and data collection methods, see supplementary material.

### Beliefs about capabilities

Beliefs about capabilities was the most frequently reported domain appearing in 13 studies and was predominantly facilitator orientated. Qualitative evidence revealed that participants’ perceived competence [27], self-confidence [27, 47, 49], self-efficacy [27, 42, 46, 47, 49, 51, 54], positive experiences in symptom relief and skills developed from intervention participation [42, 47] enabled the maintenance of PA behaviour. The findings illustrated how skill acquisition and fostered self-confidence reinforced motivation to maintain PA behaviour despite physical limitations or experiences with pain [44, 46, 47].

“*Although I experience the same level of pain*,* I have learned to continue with my activities and I realise that I achieve more because of that*“ [54].

Quantitative studies and quantitative components of mixed-methods studies (*n* = 7) [40, 41, 43, 45, 48, 50, 53] reinforced this narrative. Facilitators included greater self-efficacy and the perception of exercises as easy to perform [48, 53]. Moreover, barriers were associated with low self-discipline and lower levels of self-confidence [44, 48, 53]. Collectively, these results indicate this domain as a central mechanism underpinning PA behaviour maintenance for those with hip and/or knee OA.

### Environmental context and resources

Environmental context and resources appeared in 12 studies and was predominantly barrier orientated. This domain described changes in health status [42, 46], lack of access to resources and ongoing support [27, 41] as significantly impacting participants’ ability and motivation to maintain PA behaviour. Contextual barriers such as time constraints [42, 49] and competing priorities such as taking care of others [42, 44] often led to a lapse in maintaining PA behaviour. Conversely, resources from the formal intervention (e.g., written manuals and exercise bands) acted as a facilitator supporting the positive regulation of PA behaviour [44, 46, 47, 49].

“*If you had any problem*,* you had a take home manual*,* if you go through that*,* that would help you…if you forgot the procedure*,* you know*,* the right way to stand – or whatever –*” [47].

Quantitative findings reinforced this, with environmental limitations such as lack of time [40, 41, 43, 45, 53], sequential PA programme duration [48, 53] and a lack of individualisation of exercises [53] the most prevalent barriers. Integration of evidence highlighted the need for greater environmental resources and opportunities for shaping both opportunity and motivation to maintain PA behaviour.

### Memory, attention and decisional processes

This domain was reported in 10 studies [27, 40–44, 47, 49, 53, 54] and reflected cognitive processes such as memory and recall, prioritisation, decision-making and action planning. This domain was predominantly barrier orientated (one facilitator quotation reported qualitatively; none reported quantitatively). Qualitative evidence revealed that participants often made a cognitive decisional process to reduce their level of PA as a result of no longer experiencing pain or discomfort [44, 49, 54], changes in time or lifestyle pressure [44, 47], forgetting to perform PA as a result of cognitive overload or tiredness [27], and finally, following changes in capacity to exercise due to the management of other conditions such as diabetes or low-back pain [27, 42]. The only facilitator reported highlighted that decision-making to adapt the PA programme enabled participants to navigate to more appropriate exercises [44].

“*Probably just time and work and you just sort of forget you go ‘oh I’ll do it later’ and then later comes and you forget don’t you?*” [27].

Quantitative findings echoed the qualitative evidence regarding barriers with cognitive overload (e.g. memory recall – forgetting) apparent in one study [40] and low energy or tiredness appearing as a barrier in four studies [40, 41, 43, 53] accounting for 19.54% of the total study sample.

### Social influences

Social influences appeared in nine studies [27, 42–44, 46–48, 53, 54], and was predominantly facilitator orientated however; persistent barriers portrayed discrepancies across studies in relation to desire and availability of social support. The findings highlighted that factors such as social encouragement and accountability shaped the maintenance of PA behaviours [27, 42, 47, 54]. These appeared in an array of settings across studies from telephone-linked communication with participants [47] to family support in home settings [27, 42] and peer and professional support as part of group activity [27]. One study emphasised that developing strong therapeutic relationships encouraged the maintenance of PA behaviour [27]. Conversely, lack of social support [46, 47], feelings of alienation or negative social pressures were perceived as barriers [46]. Subsequently, it was found that many participants desired more group activity following the formal intervention and was considered a significant factor for promoting the long-term maintenance of PA behaviour [46, 47].

“*I missed the group. Maybe sometimes we could have*,* during the interim*,* have one group study again*,* come in and see how everybody’s doing*,* and then go back. So in between the phone calls*,* every now and then there’s… I don’t know*,* quarterly or whatever*,* just come in that one time. See who’s havin’ any difficulties*,* and how you can change that”* [47].

Quantitative findings revealed this domain appeared as a facilitator in three studies [43, 48, 53] and as a barrier in two studies [43, 53]. These findings complimented the qualitative findings with lack of social support and lack of an exercise partner identified as barriers [43, 53]. Facilitators included support from physiotherapist, family, friends [48, 53] and availability of an exercise partner [53]. Overall, social influences from peers, professionals and family significantly influenced the motivation to maintain PA behaviour for those living with hip and knee OA.

### Beliefs about consequences

Beliefs about consequences appeared in nine studies [27, 40–44, 49, 52, 53] and encompassed perceptions of both positive and negative outcomes of PA behaviour. Interestingly, quantitative findings indicated this domain being primarily barrier orientated; however, qualitative findings alluded to its role in enabling the maintenance of PA behaviour. The evidence revealed that improved function [27], anticipated health benefits, and preventing the deterioration of OA condition [27, 42] acted as facilitators for maintaining PA behaviour. Fear of, or exacerbation of pain [42] alongside the fear of exacerbating comorbid health conditions such as hip bursitis [44] were considered barriers to maintaining PA behaviour.

“*Having a number of health issues that need exercise. If I don’t do it*,* I will regret it*” [42].

Quantitatively; barriers mirrored those displayed in the qualitative findings. Barriers reported were pain before, during or after exercise [40, 42, 52, 53], and uncertainty about knowledge and/or the benefits of exercise [43, 53].

### Physical activity maintenance for osteoarthritis (PAMA) conceptual map

**Fig. 3:**
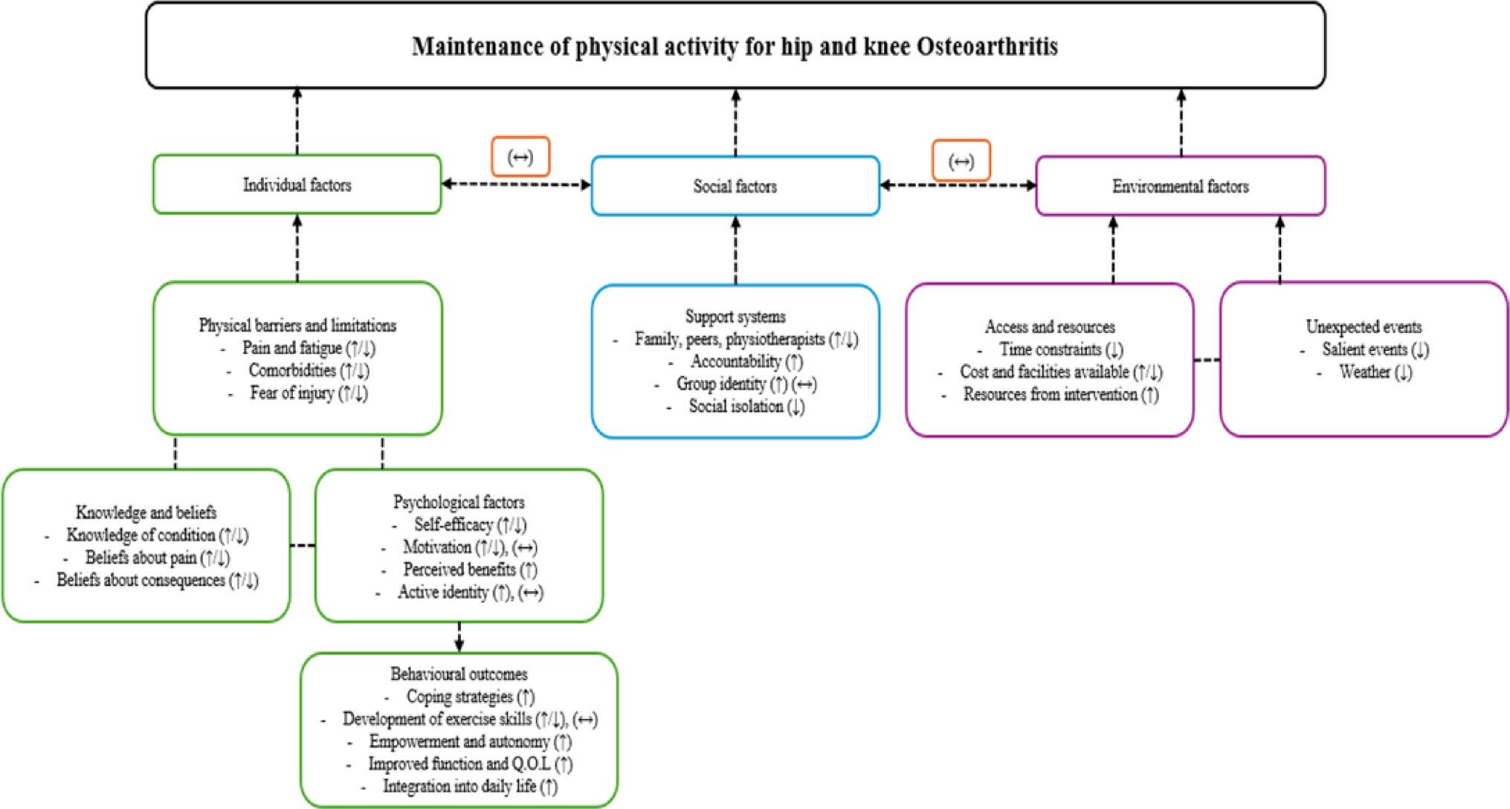
Physical Activity Maintenance for osteoArthritis (PAMA) Conceptual Map. Facilitators are indicated using ↑, barriers are indicated using ↓ and feedback loops are indicated using ⇿.

The findings of this review were subsequently mapped in the development of the PAMA conceptual map (Fig. 3), facilitating a valuable tool for synthesising and illustrating the factors influencing the maintenance of PA behaviour. This map demonstrated that PA behaviour maintenance is multifactorial and can be shaped by the dynamic interactions between individual, social and environmental factors. Within these categories, key characteristics influencing PA behaviour maintenance were identified, including, physical barriers and limitations, knowledge and beliefs, psychological factors, behavioural outcomes, support systems, access and resources and unexpected events. The conceptual map was further refined through an iterative process, and each individual characteristic was classified as either a barrier, facilitator or both based on the representative findings across all 16 included studies. This integrative process identified that factors such as integration of PA into daily life (habit formation), resources from a previous intervention, coping strategies and feelings of accountability were central to enable the maintenance of PA behaviour. Barriers such as feelings of social isolation, time constraints and salient/critical events such as immediate caring responsibilities often hindered the maintenance of PA behaviour. Several studies reported that specific factors such as living with an additional comorbidity acted as both a barrier and a facilitator. Individuals living with a comorbid condition such as diabetes [27, 52], glaucoma [42] and Low Back Pain (LBP) [44] often associated feelings of fatigue or fear of worsening their condition should they continue with PA, acting as a barrier. In other instances, PA behaviour was associated as a mechanism to help manage comorbid conditions [42], acting as a facilitator (Table 3).

**Table 2:** Summary of studies reported under each TDF domain with included barriers, facilitators and exemplar illustrative quotes

TDF Domain	No. of studies (Qualitative)	No. of studies (Quantitative)	No. of studies (mixed-methods)	Total (*n* = 16)	Studies reporting as a facilitator	Studies reporting as a barrier	Exemplar illustrative quote (from qualitative synthesis)
Knowledge	(n = 1) Moore et al., 2020 [27]	(n = 1) Stanton et al., 2020 [48]	(n = 5) Cheung et al., 2022 [42], Hinman et al., 2023 [44], Tan et al., 2025 [49], Hammer et al., 2015 [51], Matile et al., 2024 [53]	(n = 7)	(n = 7) Moore et al., 2020 [27], Cheung et al., 2022 [42], Hinman et al., 2023 [44], Stanton et al., 2020 [48], Tan et al., 2025 [49], Hammer et al., 2015 [51], Matile et al., 2024 [53]	(n = 1) Matile et al., 2024 [53]	“*Now I know how much it means to train regularly*,* where all the muscles that are important to the hip gets going*” (Hammer et al., 2015 [51]) (Facilitator)
Skills	(*n* = 1) Ledingham et al., 2019 [47]	(*n* = 0)	(*n* = 2) Cheung et al., 2022 [42], Matile et al., 2024 [53]	(*n* = 3)	(*n* = 3) Cheung et al., 2022 [42], Ledingham et al., 2019 [47], Matile et al., 2024 [53]	(*n* = 1) Matile et al., 2024 [53]	“*I combined yoga with physical therapy.. I do most of my yoga in bed before I get up because it is hard to get down on the floor*” (Cheung et al., 2022 [42]) (Facilitator)
Social/professional role and identity	(*n* = 1) Moore et al., 2020 [27]	(*n* = 0)	(*n* = 1) Cheung et al., 2022 [42]	(*n* = 2)	(*n* = 2) Moore et al., 2020 [27], Cheung et al., 2022 [42]	N/A*	“*I am very much of a habit person; I have exercise scheduled into my life*” (Cheung et al., 2022 [42]) (Facilitator)
Beliefs about capabilities	(*n* = 4) Moore et al., 2020 [27], Lawford et al., 2023 [46], Ledingham et al., 2019 [47], Veenhof et al., 2006 [54]	(*n* = 3) Bennell et al., 2020 [40], Hinman et al., 2020 [43], Stanton et al., 2020 [48]	(*n* = 6) Cheung et al., 2022 [42], Hinman et al., 2023 [44], Tan et al., 2025 [49], Wainwright et al., 2020 [50], Hammer et al., 2015 [51], Matile et al., 2024 [53]	(*n* = 13)	(*n* = 13) Moore et al., 2020 [27], Bennell et al., 2020 [40], Cheung et al., 2022 [42], Hinman et al., 2020 [43], Hinman et al., 2023 [44], Lawford et al., 2023 [46], Ledingham et al., 2019 [47], Stanton et al., 2020 [48], Tan et al., 2025 [49], Wainwright et al., 2020 [50], Hammer et al., 2015 [51], Matile et al., 2024 [53], Veenhof et al., 2006 [54]	(*n* = 8) Moore et al., 2020 [27], Bennell et al., 2020 [40], Cheung et al., 2022 [42], Hinman et al., 2020 [43], Stanton et al., 2020 [48], Tan et al., 2025 [49], Hammer et al., 2015 [51], Matile et al., 2024 [53]	“*The more exercise I do*,* the more I do with it*,* I think the better it’s gonna be*,* rather than nursing it*,* keep doing it and keep exercising it and so that’s what I do”* (Moore et al., 2020 [27]) (Facilitator) “*Probably because my performance is nothing to write home about…. My wife has told me that she really wanted to [exercise]*,* and that we could do it together*,* but I’m not too keen on that*,* because she’s too mobile*” (Hammer et al., 2015 [51]) (Barrier)
Optimism	(*n* = 1) Ledingham et al., 2019 [47]	(*n* = 2) Bennell et al., 2020 [40], Stanton et al., 2020 [48]	(*n* = 3) Cheung et al., 2015 [41], Hammer et al., 2015 [51], Matile et al., 2024 [53]	(*n* = 6)	(*n* = 2) Cheung et al., 2015 [41], Hammer et al., 2015 [51]	(*n* = 5) Bennell et al., 2020 [40], Cheung et al., 2015 [41], Ledingham et al., 2019 [47], Stanton et al., 2020 [48], Matile et al., 2024 [53]	“*I thought that it would be a good thing if I could strengthen some muscles. That would make things easier and maybe delay the need for surgery and so on. Now I’m 100% certain about tha*t” (Hammer et al., 2015 [51]) (Facilitator)
Beliefs about consequences	(n = 1) Moore et al., 2020 [27]	(n = 3) Bennell et al., 2020 [40], Hinman et al., 2020 [43], Desai et al., 2014 [52]	(n = 5) Cheung et al., 2015 [41], Cheung et al., 2022 [42], Hinman et al., 2023 [44], Tan et al., 2025 [49], Matile et al., 2024 [53]	(n = 9)	(n = 3) Moore et al., 2020 [27], Cheung et al., 2022 [42], Tan et al., 2025 [49]	(n = 8) Bennell et al., 2020 [40], Cheung et al., 2015 [41], Cheung et al., 2022 [42], Hinman et al., 2020 [43], Hinman et al., 2023 [44], Tan et al., 2025 [49], Desai et al., 2014 [52], Matile et al., 2024 [53]	“*I thought it [exercise] was doing me OK but then no*,* I just couldn’t deal with it anymore…I ended up having to have injections in my hips afterwards. Because I do have bursitis in my hips*,* so it actually created more problems for me*” (Hinman et al., 2023 [44]) (Barrier)
Reinforcement	(n = 1) Ledingham et al., 2019 [47]	(n = 0)	(n = 3) Cheung et al., 2015 [41], Cheung et al., 2022 [42], Hammer et al., 2015 [51]	(n = 4)	(n = 3) Cheung et al., 2015 [41], Cheung et al., 2022 [42], Hammer et al., 2015 [51]	(n = 1) Ledingham et al., 2019 [47]	“*…a suggestion might be to have like a reunion… Do the exercises*,* …I guess*,* hearing people’s experiences with the exercises*,* seeing if we were doing them right*,* tweaking them*,* you know*,* getting some feedback*” (Ledingham et al., 2019 [47]) (Barrier)
Intentions	(n = 0)	(n = 0)	(n = 1) Matile et al., 2024 [53]	(n = 1)	(n = 1) Matile et al., 2024 [53]	N/A*	N/A**
Goals	(n = 1) Veenhof et al., 2006 [54]	(n = 1) Hinman et al., 2020 [43]	(n = 1) Cheung et al., 2022 [42]	(n = 3)	(n = 3) Cheung et al., 2022 [42], Hinman et al., 2020 [43], Veenhof et al., 2006 [54]	N/A*	“ *I really know these exercises have beneficial effects and that motivates me to continue with my exercises. The main motivation to do all this is to prevent an operation to get a new hip*” (Veenhof et al., 2006 [54]) (Facilitator)
Memory, attention and decisional processes	(n = 3) Moore et al., 2020 [27], Ledingham et al., 2019 [47], Veenhof et al., 2006 [54]	(n = 2) Bennell et al., 2020 [40], Hinman et al., 2020 [43]	(n = 5) Cheung et al., 2015 [41], Cheung et al., 2022 [42], Hinman et al., 2023 [44], Tan et al., 2025 [49], Matile et al., 2024 [53]	(n = 10)	(n = 1) Hinman et al., 2023 [44]	(n = 10) Moore et al., 2020 [27], Bennell et al., 2020 [40], Cheung et al., 2015 [41], Cheung et al., 2022 [42], Hinman et al., 2020 [43], Hinman et al., 2023 [44], Ledingham et al., 2019 [47], Tan et al., 2025 [49], Matile et al., 2024 [53], Veenhof et al., 2006 [54]	“*Probably just time and work and you just sort of forget you go ‘oh I’ll do it later’ [exercise] and then later comes and you forget don’t you?*” (Moore et al., 2020 [27]) (Barrier) “*For the first few months*,* yes. But after that*,* I almost forget about it [exercise] because there’s no pain*,* seems like there’s no motivation to do the exercises*” (Tan et al., 2025 [49]) (Barrier)
Environmental context and resources	(n = 3) Moore et al., 2020 [27], Lawford et al., 2023 [46], Ledingham et al., 2019 [47]	(n = 4) Bennell et al., 2020 [40], Hinman et al., 2020 [43], Kawi et al., 2015 [45], Stanton et al., 2020 [48]	(n = 5) Cheung et al., 2015 [41], Cheung et al., 2022 [42], Hinman et al., 2023 [44], Tan et al., 2025 [49], Matile et al., 2024 [53]	(n = 12)	(n = 5) Cheung et al., 2022 [42], Hinman et al., 2023 [44], Lawford et al., 2023 [46], Ledingham et al., 2019 [47], Tan et al., 2025 [49]	(n = 12) Moore et al., 2020 [27], Bennell et al., 2020 [40], Cheung et al., 2015 [41], Cheung et al., 2022 [42], Hinman et al., 2020 [43] Hinman et al., 2023 [44], Kawi et al., 2015 [45], Lawford et al., 2023 [46], Ledingham et al., 2019 [47], Stanton et al., 2020 [48], Tan et al., 2025 [49], Matile et al., 2024 [53]	“ *I was a pretty physically active person in different sports*,* but.. a change occurred in my health in February*” (Cheung et al., 2022 [42]) (Barrier) “*The information that we were given through the booklets and through the website was extensive and easy to read and easy to look at and easy to follow*” (Lawford et al., 2023 [46]) (Facilitator)
Social influences	(n = 4) Moore et al., 2020 [27], Lawford et al., 2023 [46], Ledingham et al., 2019 [47], Veenhof et al., 2006 [54]	(n = 2) Hinman et al., 2020 [43], Stanton et al., 2020 [48]	(n = 3) Cheung et al., 2022 [42], Hinman et al., 2023 [44], Matile et al., 2024 [53]	(n = 9)	(n = 8) Moore et al., 2020 [27], Cheung et al., 2022 [42], Hinman et al., 2020 [43], Hinman et al., 2023 [44], Ledingham et al., 2019 [47], Stanton et al., 2020 [48], Matile et al., 2024 [53], Veenhof et al., 2006 [54]	(n = 5) Hinman et al., 2020 [43], Lawford et al., 2023 [46], Ledingham et al., 2019 [47], Matile et al., 2024 [53], Veenhof et al., 2006 [54]	“*I feel like I’ve self-destructed. I felt like once the program ended I probably—I really needed someone just doing a constant check-in with me…*” (Lawford et al., 2023 [46]) (Barrier) “*My daughter told me*,* “Mom you need to do yoga*,* you need to do yoga*” (Cheung et al., 2022 [42]) (Facilitator)
Emotion	(*n* = 0)	(*n* = 1) Hinman et al., 2020 [43]	(*n* = 4) Cheung et al., 2015 [41], Cheung et al., 2022 [42], Hinman et al., 2023 [44], Hammer et al., 2015 [51]	(*n* = 5)	(*n* = 3) Cheung et al., 2022 [42], Hinman et al., 2023 [44], Hammer et al., 2015 [51]	(*n* = 5) Cheung et al., 2015 [41], Cheung et al., 2022 [42], Hinman et al., 2020 [43], Hinman et al., 2023 [44], Hammer et al., 2015 [51]	“*Mentality*,* depressed moods/depression.. being alone*” (Cheung et al., 2022 [42]) (Barrier) “*I like [yoga in the water] so much that it motivates me to get out early in the morning*,* and I feel great*” (Cheung et al., 2022 [42]) (Facilitator)
Behavioural regulation	(*n* = 0)	(*n* = 0)	(*n* = 4) Cheung et al., 2022 [42], Hinman et al., 2023 [44], Tan et al., 2025 [49], Matile et al., 2024 [53]	(*n* = 4)	(*n* = 4) Cheung et al., 2022 [42], Hinman et al., 2023 [44], Tan et al., 2025 [49], Matile et al., 2024 [53]	(*n* = 1) Matile et al., 2024 [53]	“ *I felt*,* as I worked on the exercises – like religiously*,* doing them every second day*” (Hinman et al., 2023 [44]) (Facilitator) “*Change your habit…you have to be consistent… if you are not consistent*,* then it would not be able to help you improve. So*,* we learn to be consistent and what to do when you got this problem…. everybody is the same*,* no time*,* so must make time to do”* (Tan et al., 2025 [49]) (Facilitator)

*TDF* Theoretical domains framework;

*no studies reported a barrier under this domain

**,no qualitative studies appeared under this domain

**Table 3:** Qualitative themes and associated TDF domains with supported illustrative quotes

Theme	Barrier/Facilitator	TDF domain(s)	Summary of meaning and interpretation	Illustrative quote	Studies presenting this theme
Autonomy and independence	Facilitator	Beliefs about capabilities; Reinforcement	Participants valued PA as a means of maintaining independence and lifestyle activities, reinforcing internal rewards and confidence — consistent with *reinforcement* and *beliefs about capabilities* domains.	*“[I] could do more work in the house*,* and OK*,* maybe go out and take my grandson*,* to the park by myself. No need to wait for my husband. If my leg is not strong enough*,* I have to wait for my husband*,* cause I’m afraid that – he’s [grandson] running around*,* [I] won’t be able to catch him”* (Ledingham et al., 2019 [47]) “*Helps [you] keep up with the activities that you enjoy*” (Cheung et al., 2022 [42])	Cheung et al., 2022 [42], Ledingham et al., 2019 [47], Tan et al., 2025 [49]
Motivations, goals and emotions	Facilitator	Behavioural Regulation; Emotion; Goals; Optimism; Social Influences; Social/Professional role and identity; Reinforcement;	Motivation to maintain PA stemmed from goal setting, emotional responses, benefits, optimism, and social accountability. These reflect multiple TDF domains evolving around motivation and *behavioural regulation*.	“*The thing is you do the exercise ‘cause you feel that you don’t want to let the other person down. You know you do them ‘cause in the first instance you think*,* ‘Oh that’s going to do me good*,* it’s going to yeah’*,* but also there’s a secondary thing there you think*,* ‘Oh he’s gone out of his way to explain these things to me and shown me what to do it’s only fair that I do them so at least I can tell him what sort of effect its having the next time I meet him’*,* you know*” (Moore et al., 2020 [27]) “*We all like to get a pat on the back*,* so it is important*” (Hammer et al., 2015 [51])	Moore et al., 2020 [27], Cheung et al., 2015 [41], Cheung et al., 2022 [42], Hinman et al., 2023 [44], Lawford et al., 2023 [46], Ledingham et al., 2019 [47], Tan et al., 2025 [49], Hammer et al., 2015 [51]
Social and external support	Facilitator	Reinforcement; Social Influences	Social and professional support, group belonging, and encouragement from peers and practitioners facilitated accountability and sustained motivation, fitting within the *social influence* and *reinforcement* domains.	“*My wife. She’d say sometimes*,* ‘I haven’t seen you do your exercises’. And it may be that I’d done them when she wasn’t around*,* and I’d say*,* ‘Oh*,* yeah*,* I did them this morning*,*’ or*,* ‘I did them*,*’ you know*,* whatever. And then I’d go*,* another time I’d go [tuts]*,* ‘Yeah*,* fair cop*,* guv*,*’ you know and off we go. So*,* having someone to know what the regime is…*” (Moore et al., 2020 [27]) “*[D]o it with people—if you have a companion to exercise you’re more motivated*” (Cheung et al., 2022 [42]) “*Knowing that I’m gonna get that phone call!*” (Ledingham et al., 2019 [47])	Moore et al., 2020 [27], Cheung et al., 2022 [42], Ledingham et al., 2019 [47]
Skills, confidence and ability	Facilitator	Beliefs about capabilities; Skills	Participants developed and applied practical skills from the intervention, reinforcing confidence and their perceived ability to perform PA independently — aligning with *beliefs about capabilities* and *skills* of the TDF.	“*so the fact that I know I can do it is quite a motivator now…I think I now have the confidence and knowledge and understanding to be able to improve my health again…*” (Lawford et al., 2023 [46]) “*It got me where I had a wide variety of different exercises that I could do and I felt supported and I knew what I needed to do*,* so I didn’t really need more [physio consults]*” (Hinman et al., 2023 [44])	Cheung et al., 2022 [42], Hinman et al., 2023 [44], Lawford et al., 2023 [46], Ledingham et al., 2019 [47], Wainwright et al., 2020 [50], Veenhof et al., 2006 [54]
Resources and environment	Facilitator	Behavioural regulation; Environmental context and resources;	Access to resources (equipment, facilities, workbooks, guidance) and ability to monitor progress supported PA behaviour maintenance. Reflects environmental enablers and self-regulatory behaviours facilitating the maintenance of PA behaviour.	“*If you had any problem*,* you had a take home manual*,* if you go through that*,* that would help you…if you forgot the procedure*,* you know*,* the right way to stand – or whatever –*” (Ledingham et al., 2019 [47]) “*that was kind of a good little challenge to have your little [activity tracker] on your arm and see how many steps and you know if you need to go for an extra walk*,* well*,* I would*” (Hinman et al., 2023 [44])	Cheung et al., 2022 [42], Hinman et al., 2023 [44], Lawford et al., 2023 [46], Ledingham et al., 2019 [47], Tan et al., 2025 [49]
Knowledge and beliefs	Facilitator/Barrier	Beliefs about capabilities; Beliefs about consequences; Emotion; Environmental context and resources; Knowledge; Memory, attention and decisional processes; Optimism; Reinforcement	Participants’ knowledge and beliefs helped understand the role of exercise in managing OA and confidence in ability, while misconceptions about pain and low self-efficacy hindered participation. These patterns corresponded to an array of domains under the TDF however, primarily linked to *knowledge*, *beliefs about capabilities*, and *emotion* domains of the TDF.	“*Because it was a knee pain thing that I’m doing*,* I thought of the weight loss as a bonus. I was concentrating on getting the leg right — It would give me an opportunity to try an alternative to surgery*,* so I kept on track for that…Then eventually the knee was getting really good and the weight loss was happening so it kept me motivated to keep on going*” (Lawford et al., 2023 [46]) “…*but you can worry about – am I doing the wrong thing in order to make it worse? You’re not always sure […] ‘Cause the Doctor’ll say*,* ‘Don’t put any pressure on your knee*,*’ but if you’re walking*,* that’s some pressure. ‘Don’t do things if they hurt*,*’ then the physio might say*,* ‘Well*,* you’re not going to damage your knee anymore*,*’ but I mean you only do some things so often until you think*,* ‘Well*,* what’s the point*,*’ if it’s hurting you*,* you’re not going to; you do stop*” (Moore et al., 2020 [27])	Moore et al., 2020 [27], Cheung et al., 2015 [41], Cheung et al., 2022 [42], Hinman et al., 2023 [44], Lawford et al., 2023 [46], Ledingham et al., 2019 [47], Tan et al., 2025 [49], Hammer et al., 2015 [51], Veenhof et al., 2006 [54]
Psychological/emotional factors	Barrier	Beliefs about capabilities; Emotion	Emotional strain (e.g., stress, low mood) and poor self-efficacy hindered PA behaviour maintenance. These experiences align with affective and *beliefs about capabilities* -related domains capturing how *emotional* responses impact the maintenance of PA behaviour.	“*I persevered with it until a couple of months ago*,* because I had a lot of bad news in the family and things – stress just took over*” (Hinman et al., 2023 [44]) “*Another thing I want to tell you.you must stop me if it’s not on the agenda. that resulted in a decrease in physical activity. Pain has increased more and more – I don’t know if you can imagine how it is to be confronted with things that you want to do but are unable to do. That is mentally stressful*” (Hammer et al., 2015 [51])	Moore et al., 2020 [27], Cheung et al., 2022 [42], Hinman et al., 2023 [44], Tan et al., 2025 [49], Hammer et al., 2015 [51]
Capabilities and skills	Barrier	Beliefs about capabilities	Participants questioned their ability and competence to perform PA correctly, reflecting perceived *capability* and confidence constructs central to this domain	“*I question my own accuracy*” (Cheung et al., 2022 [42]) “*…stopped doing most of the floor yoga. getting up and down from the floor is more challenging*” (Cheung et al., 2022 [42])	Cheung et al., 2022 [42]
Time and lifestyle pressure	Barrier	Environmental context and resources; Memory, attention and decisional processes	Lack of time, competing demands and cognitive fatigue or tiredness limited continued PA participation. These barriers reflect *environmental constraints* and overload of cognitive responses impacting participants capacity for *decision-making*.	“*My disabled daughter continues to [need]a great deal. my husband got prostate cancer.*” (Cheung et al., 2022 [42]) “*I’m too busy. I don’t want to spend the time.*” (Cheung et al., 2022 [42]) “*Other things happened in my life that changed as well. just some of my physical activity ceased due to other issues*,* other people’s injuries*,* actually. So there was a bit of a sudden change in lifestyle*” (Hinman et al., 2023 [44])	Moore et al., 2020 [27], Cheung et al., 2022 [42], Hinman et al., 2023 [44], Ledingham et al., 2019 [47], Tan et al., 2025 [49]
Social and environmental influence	Barrier	Environmental context and resources; Social influences; Reinforcement	Social and environmental factors (e.g., lack of support, isolation post-programme, limited resources) directly affected PA continuation, aligning with domains addressing *social influence* and *contextual barriers*.	“*I’ve found over the years that my biggest stumbling block is the accountability side of it. So while on the program I’m fine*,* but as soon as I come off the program*,* it’s the fact that I’m not accountable to anybody but myself then and it’s not enough to keep me right…*” (Lawford et al., 2023 [46]) “*So I got out of the routine of doing them completely. I mean to my mind*,* I suppose it’s a situation that says if you are going to do exercises*,* you need somebody to give you the exercises*,* you need to be in a room with others. because you are in the house the environment is wrong*,* no motivation…*” (Moore et al., 2020 [27])	Moore et al., 2020 [27], Cheung et al., 2022 [42], Lawford et al., 2023 [46], Ledingham et al., 2019 [47], Veenhof et al., 2006 [54]
Positive perception of condition	Barrier	Memory, attention and decisional processes	Participants’ improved condition led to reduced PA (believing it was no longer needed), showing a *decision-making process* informed by perceived recovery, consistent with this TDF domain.	“*Because my complaints disappeared*,* I was no longer motivated to continue with the exercises and activities*” (Veenhof et al., 2006 [54]) “*I must admit*,* towards the end*,* I did flag off a little bit. Mainly because my knee was feeling so good*” (Hinman et al., 2023 [44]) “*For the first few months*,* yes. But after that*,* I almost forget about it because there’s no pain*,* seems like there’s no motivation to do the exercises*” (Tan et al., 2025 [49])	Hinman et al., 2023 [44], Tan et al., 2025 [49], Veenhof et al., 2006 [54]

## Discussion

This systematic review is the first to synthesise the existing quantitative and qualitative evidence on the barriers and facilitators to maintaining PA behaviour following exercise interventions for hip and knee OA. While most existing evidence focuses on initiation or adherence to OA specific exercise [26, 55], the factors underpinning long-term PA behaviour maintenance remain under prioritised and this review endeavoured to address that. The integration of a mixed-methods approach facilitated the quantitatively reported barriers and facilitators to be integrated with the in-depth lived experiences provided through the qualitative evidence; facilitating a comprehensive understanding of the individual, social and environmental factors influencing PA behaviour maintenance for those living with hip and knee OA.

The TDF in this study provided a robust structure for the integration and synthesis of diverse evidence across heterogenic methodologies, ensuring the interpretations in this review were theoretically informed to guide the understanding of behaviour as deemed appropriate by Cane et al. [56]. Prominent domains in this review revealed that *beliefs about capabilities* such as participants’ self-confidence [27, 47, 49] and self-efficacy [27, 42, 46, 47, 49, 51, 54], *environmental context and resources* such as competing priorities (e.g. taking care of others) [42, 44] and *social influences* such as social encouragement and accountability [27, 42, 47, 54] significantly influenced the maintenance of PA behaviour. These domains interacted dynamically to inform the understanding of behaviour change rather than operating as isolated determinants. In integrating and exploring these dynamic interactions, a synthesis tool was developed via the PAMA conceptual map (Fig. 3), enabling the factors influencing PA behaviour maintenance to be further explored. This showed that motivation to perform PA often reinforced confidence in PA participation, reinforcing motivation to maintain PA behaviour. Conversely, time constraints [27, 41, 42, 44, 49, 53] and feelings of social isolation [27, 43, 46, 47, 51, 53, 54] often acted as barriers for maintaining PA behaviour. The PAMA conceptual map, therefore, acts as a useful tool for synthesising the diverse evidence influencing long-term PA behaviour. The suggestions from this review reflect that PA behaviour maintenance operates dynamically whereby individual, social and environmental factors interact continuously, offering a foundation for future PA-based longitudinal interventions.

These findings align with broader behaviour change models for self-management; whereby self-regulation, social support, reinforcement and outcome expectations are central for behaviour change such as social cognitive theory, the Capacity, Opportunity, Motivation – Behaviour (COM-B) model and in line with the Transtheoretical model (TTM) [23, 57, 58]. Furthermore, a recent systematic review of health behaviour change models revealed that once motivation to sustain an activity decrease, the need for self-regulation increases [59]. In 2024, Carvalho et al. [60] expanded on this, identifying comparable determinants of maintaining PA behaviour and healthy eating habits in type two diabetes; whereby social support, habit formation, access to appropriate resources and fear of negative consequences were key features for maintaining behaviour.

However, specific and unique factors emerged in the OA population. Fatigue/tiredness [40, 41, 43, 53], pain variability [40–42, 51–53], and salient events such as surgery or fluctuations in other conditions emerged as barriers complicating sustained PA behaviour [27, 42, 44]. In some instances, participants chose to disengage with PA following improvements in their OA condition and no longer experiencing the same level of pain [44, 49, 54]. These periods may act as clinically significant ‘risk periods’ where relapses in behaviour may occur due to exacerbations in pain, fluctuations in comorbid conditions and salient events may disrupt routine. As per the TTM, it is during early maintenance (6–12-months) where relapse is most common [23] and thus, understanding and action planning for factors influencing ‘risk periods’ is significant for optimising long-term PA behaviour. Participants frequently described strong therapeutic relationships and individualised or adapted self-management strategies were essential facilitators for maintaining PA behaviour [27, 42]. Furthermore, it must be noted that participants in this study were predominantly female (73.7%), aligning with current global prevalence rates whereby females account for 60% of all OA affected cases with an increase seen in those aged ≥ 40 years old [61].

While many similarities exist between initiation, adherence and maintenance of PA behaviour such as self-efficacy, environmental factors, outcome expectations, and support; the current synthesis revealed some key distinctions. In comparison with a systematic review on adherence to PA behaviour for those with hip and knee OA [26], the current synthesis revealed a stronger role of developing autonomy, habit formation and the provision of ongoing support as key for maintaining long-term PA behaviour. Participants that maintained PA behaviour notably integrated PA into their daily lives for various self-management implications such as reducing pain, facilitating daily activities, and building confidence in managing their condition. These novel findings for PA behaviour maintenance instil the importance of fostering motivation and self-determination as opposed to relying on external accountability that is not always feasible. The evidence synthesised in this review therefore suggests several key principles for designing future interventions to support the maintenance of PA behaviour for those with hip and knee OA. Interventions should adopt a longitudinal lens whereby structured support is offered initially, however transition toward fostering autonomy and independence at the maintenance phase. This process should incorporate multiple complimentary BCTs such as action planning for fluctuations in pain and health status, consolidation of habit formation integrated into daily routine and supporting the development of self-regulation and self-efficacy through techniques such as verbal persuasion and positive reinforcement.

### Strengths and limitations

The strengths of this systematic review included its novel mixed-methods design, the TDF theoretically driven synthesis, and the development of an evidence synthesis tool via the integration of the PAMA conceptual map that illustrates the dynamic factors affecting long-term PA behaviour. Integration of quantitative and qualitative evidence facilitated the triangulation of findings, enabling an understanding of the underlying contextual mechanisms at which the quantitative barriers and facilitators were reported. The use of the TDF provided a theoretical basis for understanding behaviour change, allowing the findings to be transformed from interpretive results into potential actionable targets for future interventions.

However, several limitations exist in this study and should be acknowledged. A central limitation of this review is that studies differed in their exploration of barriers and facilitators to maintaining PA behaviour with some exploring adherence to exercises and others exploring broader PA behaviour such as lifestyle-based PA. This causes complications in the overall synthesis of evidence, and the subjective interpretation of the term ‘maintenance’ must be acknowledged. In this study the broader conceptualisation of PA behaviour and bridging the gap from structured exercise towards maintaining daily PA behaviour was considered the primary focus and thus, these results were discussed collectively. Population demographics were predominantly female, aligning with global prevalence rates however; certain factors such as reduced physical function and reduced weekly PA have been reported more frequently in females [61], and should be considered when interpreting the findings of this study. Duration of follow-up varied significantly across studies ranging from six-months to five years. This disparity causes complications in making accurate comparisons however still falls within the TTM stages of change for ‘maintenance’ [23]. Quantitative evidence was heavily driven by self-report questionnaires and experienced a mix of closed-text multiple choice questions with fewer open-text questions, leading to a predominantly a priori set of barriers and facilitators. The mixed-methods approach enriched this understanding by capturing the lived-experiences; engrossing a broader set of barriers and facilitators however, synthesising this data inevitably involves some subjective interpretation and must be acknowledged.

### Implications, future research and conclusion

These findings highlight the need for strategies that optimise the maintenance of PA behaviour for those with hip and/or knee OA. For healthcare professionals delivering exercise interventions, it is pivotal that behavioural strategies are implemented prior to discharge for promoting long-term PA behaviour. Key messages derived from this review suggest focusing on supporting the transition from supervised exercise towards sustaining long-term lifestyle-based PA behaviour. This may be achieved through encouraging and fostering self-efficacy, discussing adaptive coping strategies and action planning for fluctuations in PA behaviour. Furthermore, a staged reduction in contact could be pivotal for providing ongoing support while also enhancing participants’ self-confidence, autonomy and independence. Behaviour change techniques such as goal-setting, feedback and social support are significant in the early stages however, transitioning to developing self-confidence, self-efficacy, autonomy, action planning and habit reinforcement [58, 62] alongside sequential staggered support methods such as short message services [40] could prove invaluable for maintaining PA behaviour.

Prospective longitudinal studies that integrate behaviour change for hip and knee OA should focus on the incorporation of theory driven and tested BCTs. It is known that adjuncts for PA behaviour maintenance have a greater chance of success when delivered sequentially and when multiple complimentary BCTs have been incorporated [63]. The conceptual map proposed in this review was used to synthesise the diverse evidence for maintaining PA behaviour, however, may act as a testable model for future investigations, to capture and explore the factors influencing long-term PA behaviour maintenance. The use of the TDF enables the translation of these domains to BCTs, facilitating actionable processes for the integration of BCTs in future intervention designs [64]. BCTs that target beliefs about capabilities such as verbal persuasion may help in promoting self-efficacy with techniques such as action planning through goal setting, habit formation and behavioural contracts reinforcing planned behaviour suitable for targeting environment driven barriers [58, 65].

In summary, this mixed-methods review provides the first comprehensive synthesis of barriers and facilitators to maintaining PA behaviour for those living with hip and knee OA. Through the integration of quantitative and qualitative evidence in conjunction with the TDF and the development of a conceptual map; this review sheds light on understanding the dynamic interplay of factors influencing the maintenance of PA behaviour. This systematic review builds on the existing knowledge base of adherence to PA behaviour and emphasises key differences in transitioning from exercise adherence to maintenance of PA behaviour. The insights derived from this review unfortunately do not provide the panacea for OA; however, contribute significantly to the construction of the overall paradigm for long-term hip and knee OA management.

## Supplementary Information

Below is the link to the electronic supplementary material.

Supplementary Material 1

## Data Availability

The data included in this systematic literature review is available in the article and via the online accessible supplementary material.
