# Supplementary material for: Barriers to and facilitators of maintaining physical activity for people with hip and knee osteoarthritis: a mixed-methods systematic review
Source: Rheumatol Int. 2026 Jul 1;46(7):180. doi: 10.1007/s00296-026-06230-0 (PMC13323625; doi:10.1007/s00296-026-06230-0)
Supplement: Supplementary file 1 — Supplementary Material 1 [file 296_2026_6230_MOESM1_ESM.docx]

# **Supplementary Materials**

## **Search strategy – PubMed**

**No date limit applied.**

**Date of search: 28^th^ February 2025**

**Results: 1428 records**

| **#1** | osteoarthritis, hip OR Osteoarthritis, Knee OR Osteoarthritis [MeSH Terms] | 104,520 |
| --- | --- | --- |
| **#2** | "Osteoarthritis"[Title/Abstract] OR "hip osteoarthritis"[Title/Abstract] OR "knee osteoarthritis"[Title/Abstract] OR "hip oa"[Title/Abstract] OR "knee oa"[Title/Abstract] OR "lower limb osteoarthritis"[Title/Abstract] OR "lower limb oa"[Title/Abstract] | 99,119 |
| **#3** | “Physical activity"[Title/Abstract] OR "exercis*"[Title/Abstract] OR "physical inactivity"[Title/Abstract] | 530,534 |
| **#4** | “Maintain” [Title/Abstract] OR "Maintenance"[Title/Abstract] OR "sustain*"[Title/Abstract] OR "Long-term"[Title/Abstract] OR "adher*"[Title/Abstract] | 2,863,013 |
| **#5** | #1 OR #2 | 125,137 |
| **#6** | #5 AND #3 | 7,105 |
| **#7** | #5 AND #3 AND #4 | 1,485 |
| **#8** | **#5 AND #3 AND #4**  Filters applied: English | **1,428** |

**Update of PubMed search March 2026,**

**Filters: Date limit applied (Articles published since 28-02-2025), Language: English.**

**Date of search: 25 March 2026**. **Results: 220 records**

| **#1** | osteoarthritis, hip OR Osteoarthritis, Knee OR Osteoarthritis [MeSH Terms] | 8,242 |
| --- | --- | --- |
| **#2** | "Osteoarthritis"[Title/Abstract] OR "hip osteoarthritis"[Title/Abstract] OR "knee osteoarthritis"[Title/Abstract] OR "hip oa"[Title/Abstract] OR "knee oa"[Title/Abstract] OR "lower limb osteoarthritis"[Title/Abstract] OR "lower limb oa"[Title/Abstract] | 10,668 |
| **#3** | “Physical activity"[Title/Abstract] OR "exercis*"[Title/Abstract] OR "physical inactivity"[Title/Abstract] | 42,768 |
| **#4** | “Maintain” [Title/Abstract] OR "Maintenance"[Title/Abstract] OR "sustain*"[Title/Abstract] OR "Long-term"[Title/Abstract] OR "adher*"[Title/Abstract] | 256,725 |
| **#5** | #1 OR #2 | 11,044 |
| **#6** | #5 AND #3 | 881 |
| **#7** | #5 AND #3 AND #4 | 220 |

**Supplementary table 1.** Study characteristics of included studies

| Study  Lead author (year) | Country | Aim | Population characteristics | Methods (design, follow-up duration, data collection and analysis) | Findings | MMAT quality |
| --- | --- | --- | --- | --- | --- | --- |
| Bennell (2020) [[39](#_ENREF_39)] | Australia | To evaluate whether a 24-week SMS intervention improves adherence to unsupervised home exercise in people with knee OA and obesity compared with no SMS intervention. | N = 110 participants (67.2% female)  Mean age:  SMS group (61.7 ± 6.7 years), Control group 62.9 ± 6.8 years),  Knee OA | Quantitative,  6-months  A parallel two-group superiority RCT. Primary outcomes adapted from the Exercise adherence rating scale (EARS). Automatic SMS data was also captured and barrier selection and frequency of barriers recorded. Data analysed using Chi-squared tests, t-tests, linear regression models and complete case analysis. | SMS support significantly improved adherence to home exercise compared with control (higher EARS score, more exercise days). 28% of the population asked did not report any barriers. | High (100%) |
| Cheung (2022) [[41](#_ENREF_41)] | USA | To describe and compare long-term Hatha Yoga and Aerobic strength training adherence and to identify the barriers and facilitators to long-term adherence. Furthermore, this study aimed to examine additional self-care interventions at 12-months. | N = 28 participants (100% female),  Mean age: 71.2 ± 7.8 years  Knee OA | Mixed-methods,  6- and 12-months  Qualitative: Focus group interviews lasting 90-minutes and analysed using an inductive content analysis.  Quantitative: Adherence data and frequency and type of PA collected through exercise booklets.  Results discussed using a narrative synthesis. | Average PA: 3.5 days per week. Participants used various self-care methods (Aerobic strength exercise, supplements, diets, therapy) for management of OA. | High (82.35%) |
| Cheung (2015) [[40](#_ENREF_40)] | USA | To determine yoga adherence in community older female adults with knee OA 6-months after the cessation of an 8-week yoga programme. | N = 31 participants (100% female),  Mean age: 72 ± 5.6 years, Knee OA | Mixed-methods,  6-months  Cross-sectional descriptive design using survey, interviews and video analysis.  Qualitative: Individual interviews.  Quantitative: Survey questionnaire to examine frequency and duration of yoga practice and barriers to exercise. | 91% survey response. 61% continued yoga (mostly home-based). Main motivator identified was ‘feeling better’. Barriers discussed were health problems (such as surgery), pain, time constraints, tiredness and beliefs about exercise (boring, not helpful). | Medium (73.53%) |
| Desai (2014) [[51](#_ENREF_51)] | USA | To examine the impact of telephone reinforcement (TR) on predictors of PA maintenance in older adults with OA. | N = 486 participants (86.6% female),  Mean age: 71.1 years, range (59 to 91 years)  Both hip and knee OA | Quantitative,  6-, 12-, and 18-months  Mixed-effects modelling of a randomised PA trial of negotiated maintenance contracts, supplemented by Telephone reinforcement (TR), to test impact of TR on barriers, decisional balance, and stage of change at multiple time points. | Negotiated contracts improved PA maintenance and were more cost-effective. TR increased positive perceptions but not behaviour. | High (78.57%) |
| Hammer (2015) [[50](#_ENREF_50)] | Denmark | To examine self-efficacy in relation to post-intervention PA maintenance and to identify differences between maintainers and non-maintainers of PA and the contribution of self-efficacy | N = 52 participants (69% female),  Mean age: 69 years, range (65-74 years),  Hip OA | Mixed-methods,  8-months  A parallel mixed data analysis design.  Quantitative: Survey questionnaires on level of PA, maintenance of PA and self-efficacy.  Qualitative: Semi-structured interviews and analysed using self-efficacy theory.  Results discussed using a narrative synthesis. | 60% maintained PA at 12-months; maintainers had improved Q.O.L and self-efficacy. Altruism emerged as a new theme independent of self-efficacy theory. | High (91.18%) |
| Hinman (2023) [[43](#_ENREF_43)] | Australia | Absence of Improvement with Exercise in Some Patients with Knee Osteoarthritis: A Qualitative Study of Responders and Non-responders | N = 26 participants  (68% female),  Mean age: responders (57 ± 7 years), non-responders (67 ± 9 years),  Knee OA | Qualitative,  6-months  Semi-structured interviews and analysed using a deductive thematic analysis and the findings then triangulated with quantitative data. | Responders showed empowerment; non-responders faced more comorbidities, stress, weight-related barriers and lower adherence. | High (100%) |
| Hinman (2020) [[42](#_ENREF_42)] | Australia | Does telephone-delivered exercise advice and support by physiotherapists improve pain and/or function in people with knee osteoarthritis? Telecare randomised controlled trial | N = 175 participants  (62.86% female),  Mean age: Existing service (62.5 ± 8.1 years),  Exercise advice and support group (62.4 ± 9.1 years),  Knee OA | Quantitative,  6-months  A pragmatic superiority parallel-group RCT. Difference in change (baseline minus follow-up) estimated using mixed-linear regression models. For global changes, participants 'moderately' or 'better' or much better' ('moderately more' or 'much more') were classified as improved. Intervention effects assessed under full adherence ('complier average casual effects') using a two-stage least squares instrumental variables approach. | Both groups improved in pain / function; EAS showed higher PA and self-efficacy but not statistically significant. Barriers increased by 12-months. | High (100%) |
| Kawi (2015) [[44](#_ENREF_44)] | USA | To develop and implement an online self-management program to enhance adherence, progression and maintenance of exercise. | N = 16 participants  (100% female),  Mean age: 60.9 years range (52 – 72 years),  Knee OA | Quantitative,  6-months  A quasi-experimental pilot study with two-arms: progressive walking or progressive stepping. All measures were quantitatively captured at timepoints 6-weeks and 6-months follow-up of the 10-week intervention. | 63% continued exercising ≥ 4 times per week. Walking was the most common exercise performed. | High (92.86%) |
| Lawford (2023) [[45](#_ENREF_45)] | Australia | To identify and address the barriers that exist 6-months post-intervention of a multicomponent weight loss programme for the overweight / obese population with co-existing knee OA. | N = 20 participants (50% female),  Mean age: 65 ± 9 years,  Knee OA | Qualitative,  6-months  Qualitative design using an interpretivist paradigm. Interviews designed using PPI with a sample population and conducted with whole cohort. Findings analysed using a thematic analysis based on a phenomenological framework to gather the lived experiences of the population. | Three key themes associated with maintenance of PA and diet. Sustained weight loss, empowered self-management (knowledge, resources, motivation) and challenges (loss of accountability, life events). | High (100%) |
| Ledingham (2019) [[46](#_ENREF_46)] | USA | To understand participants’ experiences, feelings and perspectives with exercise over 2 years while participating in the BOOST study and to identify factors that influenced adherence to exercise after 2 years | N = 25 participants  (84% female),  Mean age: 67 ± 6.1 years  Knee OA | Qualitative,  24-months  Semi-structured interviews analysed using a constant-comparative method of data analysis. Findings analysed using the interpretive paradigm. | High adherence was linked to self-determination, Physiotherapist supervision, social support, and knowledge of exercises. Low adherence tied to ambivalence and need for support. | High (100%) |
| Matile (2024) [[52](#_ENREF_52)] | Switzerland | To investigate GLA:D Switzerland participants’ self-reported level of PA adherence and to explore the barriers, facilitators and support needs to achieve long-term GLA:D exercise adherence. | N = 350 participants  (66.6% female),  Mean age: Quantitative (67 ± 9.3 years), Qualitative (64 ± 9.3 years),  Both hip and knee OA | Mixed-methods,  5 - 17 months  A mixed-methods study using an exploratory sequential design.  Qualitative: Semi-structured interviews and focus groups.  Quantitative: Survey developed from the qualitative interviews and administered online to GLA:D Switzerland participants. Analysis and group comparisons conducted using Fishers exact test, odds ratio and confidence intervals. | 84% met PA guidelines; most significant barriers were low motivation / self-discipline, and poor integration. Most significant facilitators were ease of exercise and visible progress. The preferred supports were shorter home sessions and continued group / physiotherapist contact. | High (88.24%) |
| Moore (2020) [[27](#_ENREF_27)] | UK | To investigate participants experiences of treatment and barriers and facilitators to exercise and general PA behaviour long-term. | N = 30 participants (50% female),  Mean age: Not specified however age for inclusion: (≥ 45 years),  Knee OA | Qualitative,  12-months  Longitudinal qualitative study. Semi-structured interviews conducted and analysed using a ‘layered approach’ thematic analysis followed by a deductive thematic analysis. | Supervision, progression, and individualisation enhanced motivation. Long-term barriers were pain, low-motivation, conflicting advice and limited support. Key facilitators identified were active identity, perceived benefits of PA, therapeutic alliance and social support. | High (100%) |
| Stanton (2020) [[47](#_ENREF_47)] | Australia | To evaluate the feasibility of conducting a clinical trial to determine the effect of adding Pain Science Education vs adding sham ultrasound to an individualised, physiotherapist-led education and walking program in people with painful knee osteoarthritis. | N = 20 participants (70% female),  Mean age: 67 ± 7.4 years,  Knee OA | Quantitative,  6-months  A feasibility study using RCT. Main outcomes were feasibility criteria, recruitment, intervention adherence and viability of wrist-worn accelerometer. | High call (97%) and workbook (78%) completion. Positive feedback but preference for more follow-up and more simplistic material resources. | Medium (71.43%) |
| Tan (2025)  [[48](#_ENREF_48)] | Singapore | To evaluate the implementation process of a community-based, multidisciplinary intervention (CONNACT) through an RCT in order to contextualise the RCT outcomes and inform implementation opportunities | N = 22 participants (63.6% female),  Mean age: 68.3 ± 6.92 Years,  Knee OA | Mixed-methods,  6-months  An embedded qualitative process evaluation of the Collaborative model of care between orthopaedics and allied health professionals in knee OA within an effectiveness-implementation hybrid RCT. Qualitative data analysed using a framework analysis. | The RCT demonstrated that improvement for primary outcome (KOOS) showed no significant differences between usual Care and CONNACT intervention group on long-term follow-up (12-months).  Secondary outcomes: CONNACT intervention group superior results in physical performance, knee satisfaction, GPE, and positive dietary changes in the short-term (post-intervention). | High  (94.12%) |
| Veenhof (2006) [[53](#_ENREF_53)] | Netherlands | To investigate the factors attributed to successfully integrating exercise into the daily lives of the OA population following a behavioural graded activity programme for hip and knee OA. | N = 12 participants (74% female),  Mean age: 70 years (range 55 – 80 years),  Both hip and knee OA | Qualitative,  15 - 20 months  Open-ended in-depth interviews conducted. Data was analysed using a grounded theory approach. Peer debriefing was used as the interim analyses were discussed by a group of researchers. | Mixed-alignment between perceived pain improvement and adherence. Half maintained PA but half did not. Barriers and facilitators discussed elsewhere. | High (100%) |
| Wainwright (2020) [[49](#_ENREF_49)] | UK | To explore whether people with hip OA who completed the CHAIN program continued to maintain exercise 5 years after study completion. | N = 83 participants  (54% female),  Mean age: 67.35 ± 8.59 years,  Hip OA | Quantitative,  4 – 6 years  A cross-sectional survey was sent to participants approximately 5 years after completion of the CHAIN intervention. Questions were multiple choice and last question was open-ended. | 96% reported improved knowledge; 93% improved self-management; 81% walked weekly and many either joined a gym or purchased bikes. 45% avoided GP follow-up and 43% of the population underwent hip surgical intervention. | Medium (71.43%)  ^[[1]](#footnote-1)^ |

**Supplementary table 2.** **Qualitative barriers for maintaining PA behaviour mapped to the TDF**

| Theme | Mapped TDF domain | Mapped TDF Constructs | Rationale / justification |
| --- | --- | --- | --- |
| Knowledge and beliefs | Beliefs about Consequences; Memory, Attention and Decision Processes; Optimism; Beliefs about Capabilities | Beliefs; Decision Making; Pessimism; Perceived Competence | Participants’ understanding, knowledge and beliefs about PA influenced their choices - some believed PA worsened pain, while others made decisions based on perceived benefit. Low optimism and self-belief indicated limited confidence in outcomes, aligning with these TDF domains |
| Capabilities and skills | Beliefs about Capabilities | Self-Confidence; Perceived Competence | Participants questioned their ability and competence to perform PA correctly, reflecting perceived capability and confidence constructs central to this domain |
| Physical health limitations | Environmental Context and Resources; Emotion; Beliefs about Consequences; Memory, Attention and Decision Processes | Salient Events; Fear; Consequences; Cognitive Overload | Physical limitations, pain, and fear of exacerbating conditions limited the maintenance of PA behaviour. These reflect contextual and emotional barriers, salient events that influenced behaviour, and cognitive responses in relation to fear. |
| Time and lifestyle pressure | Environmental Context and Resources; Memory, Attention and Decision Processes | Barriers; Salient Events; Cognitive Overload / Tiredness | Lack of time, competing demands and cognitive fatigue or tiredness limited continued PA participation. These barriers reflect environmental constraints and overload of cognitive responses impacting participants capacity for decision-making. |
| Psychological / emotional factors | Emotion; Beliefs about Capabilities | Depression; Stress; Self-Efficacy; Self-Esteem | Emotional strain (e.g., stress, low mood) and poor self-efficacy hindered PA maintenance. These experiences align with affective and capability-related domains capturing how emotional responses impact the maintenance of PA behaviour. |
| Positive perception of condition | Memory, Attention and Decision Processes | Decision Making | Participants’ improved condition led to reduced PA (believing it was no longer needed), showing a decision-making process informed by perceived recovery, consistent with this TDF domain. |
| Social and environmental influence | Social Influences; Environmental Context and Resources; Reinforcement | Social Support; Alienation; Material Resources; Reinforcement | Social and environmental factors (e.g., lack of support, isolation post-programme, limited resources) directly affected PA continuation, aligning with domains addressing social influence and contextual barriers. |

**Supplementary table 3.** **Qualitative facilitators for maintaining PA behaviour mapped to the TDF**

| Theme | Mapped TDF domain | Mapped TDF Constructs | Rationale / justification |
| --- | --- | --- | --- |
| Resources and Environment | Environmental Context and Resources; Behavioural Regulation | Resources / Material Resources; Self-Monitoring | Access to resources (equipment, facilities, workbooks, guidance) and ability to monitor progress supported PA maintenance. Reflects environmental enablers and self-regulatory behaviours facilitating the maintenance of PA behaviour. |
| Skills, confidence and ability | Skills; Beliefs about Capabilities | Skill Development; Self-Efficacy; Competence | Participants developed and applied practical skills from the intervention, reinforcing confidence and their perceived ability to perform PA independently — aligning with capability and skills constructs of the TDF. |
| Motivations, goals and emotions | Goals; Behavioural Regulation; Emotion; Optimism; Reinforcement; Social / Professional Role and Identity; Social Influences | Goal / Target Setting; Implementation Intention; Positive Affect; Habit Formation; Optimism; Reward; Social Pressure; Social Identity | Motivation to maintain PA stemmed from goal setting, emotional responses, benefits, optimism, and social accountability. These reflect multiple TDF domains evolving around motivation and behavioural regulation. |
| Knowledge and beliefs | Knowledge; Beliefs about Capabilities; Beliefs about Consequences; Emotion; Optimism; Reinforcement; Environmental Context and Resources; Memory, Attention and Decision Processes | Knowledge of Condition; Self-Efficacy; Consequences; Positive Affect; Empowerment; Decision-Making; Person × Environment Interaction | Understanding the value of PA for condition management, belief in ability, and positive emotional responses contributed to the maintenance of PA. These align with knowledge-, belief-, and emotion-based domains of the TDF. |
| Autonomy and independence | Reinforcement; Beliefs about Capabilities | Rewards / Incentives; Self-Confidence | Participants valued PA as a means of maintaining independence and lifestyle activities, reinforcing internal rewards and confidence — consistent with reinforcement and capability domains. |
| Social and external support | Social Influences; Reinforcement | Social Support; Group Identity; Power | Social and professional support, group belonging, and encouragement from peers and practitioners facilitated accountability and sustained motivation, fitting within the social influence and reinforcement domains. |

## **Development of the Physical Activity Maintenance for osteoArthritis (PAMA) Conceptual Map**

The findings in this study from the thematic analysis and using the Theoretical Domains Framework (TDF) facilitated the development of the Physical Activity Maintenance for osteoArthritis (PAMA) conceptual map. The key focus question in this study was to identify the barriers to and facilitators of maintaining PA behaviour for individuals with hip and knee OA. As proposed by [Novak and Cañas [37]](#_ENREF_37), a conceptual map should be designed by identifying the key focus question, listing the key concepts that influence the focus question, constructing a preliminary conceptual map and through an iterative process, continual review and revision of this conceptual map. [Novak and Cañas [37]](#_ENREF_37) further discuss the implementation of cross-links between concepts however as this study was primarily focused on the identifying the barriers to and facilitators of maintaining PA behaviour; a focus was placed on understanding the influence of each factor as a barrier or facilitator to the outcome (maintenance). This method was proposed by [Artinian [38]](#_ENREF_38) which enabled us to illustrate the representation and organisation of knowledge identified in this study. In line with the integrative nature of the TDF, which encompasses individual, social and environmental determinants of behaviour [[36](#_ENREF_36)], the findings of this study were grouped into three overarching categories of individual, social and environmental factors. The following tables (***Supplementary table* *4*** and ***Supplementary table* 5**) illustrate how these were interpretively mapped to form the PAMA conceptual map.

**Supplementary table 4.** Development of the PAMA Conceptual map

| **Analytical theme** | **Characteristics of theme** | **Heading** | **Factor** | **TDF mapping** |
| --- | --- | --- | --- | --- |
| Autonomy and independence | Empowerment and autonomy | Behavioural outcomes | Individual | Beliefs about capabilities |
| Capabilities and skills | Development of exercise skills | Behavioural outcomes | Individual | Skills |
| Knowledge and beliefs | Knowledge of condition | Knowledge and beliefs | Individual | Knowledge |
|  | Beliefs about pain | Knowledge and beliefs | Individual | Beliefs about consequences |
|  | Beliefs about consequences | Knowledge and beliefs | Individual | Beliefs about consequences |
| Motivations, goals and emotions | Motivation | Psychological factors | Individual | Reinforcement |
|  | Fear of injury | Physical barriers and limitations | Individual | Emotion |
|  | Active identity | Psychological factors | Individual | Social / professional role and identity |
| Physical health limitations | Pain and fatigue | Physical barriers and limitations | Individual | Emotion |
|  | Comorbidities | Physical barriers and limitations | Individual | Emotion |
| Positive perception of condition | Knowledge of condition | Knowledge and beliefs | Individual | Knowledge |
| Skills, confidence and ability | Perceived benefits | Psychological factors | Individual | Beliefs about capabilities |
|  | Improved function and Q.O.L. | Behavioural outcomes | Individual | Behavioural regulation |
|  | Coping strategies | Behavioural outcomes | Individual | Behavioural regulation |
|  | Integration into daily life | Behavioural outcomes | Individual | Behavioural regulation |
|  | Self-efficacy | Psychological factors | Individual | Beliefs about capabilities |
| Psychological / emotional factors | Fear of injury | Physical barriers and limitations | Individual | Emotion |
|  | Social isolation | Support systems | Social | Social influences |
| Social and Environmental influence | Group identity | Support systems | Social | Social influences |
|  | Social isolation | Support systems | Social | Social influences |
| Social and external support | Family, peers, physiotherapists | Support systems | Social | Social influences |
|  | Accountability | Support systems | Social | Social influences |
| Resources and environment | Cost and facilities available | Access and resources | Environmental | Environmental context and resources |
|  | Resources from intervention | Access and resources | Environmental | Environmental context and resources |
|  | Weather | Access and resources | Environmental | Environmental context and resources |
| Time and lifestyle pressure | Time constraints | Access and resources | Environmental | Environmental context and resources |
|  | Salient / critical events | Unexpected events | Environmental | Environmental context and resources |

**Supplementary table 5.** Characteristics of the PAMA Conceptual map

| **Factors** | **Headings** | **Characteristics** | **Descriptions** | **TDF Domain (Construct)** | **Facilitator and/or Barrier & exemplar quotes** | **Supporting studies** |
| --- | --- | --- | --- | --- | --- | --- |
| Individual Factors | Physical barriers and limitations | Pain and fatigue | Physical features of Pain; Pain flare-ups; reduced pain; stiffness; fatigue | **Emotion** (negative affect / Burn-out) | Pain and fatigue were often experienced as a barrier. Pain when linked to exercise and fatigue from other activities often reduced PA participation. Conversely, where PA improved pain or individuals described high energy levels was considered a facilitator.  “*Exercise really exacerbated my knee pain a lot*” [[41](#_ENREF_41)]  “*…the physical part of it is that I can be in some pain when I am on the way to the exercise-session and then I am actually pain-free when I go home. So, it’s actually quite easy and motivating to get it done*” [[50](#_ENREF_50)] | Qualitative  [[27](#_ENREF_27), [43](#_ENREF_43), [53](#_ENREF_53)]  Quantitative  [[39](#_ENREF_39), [42](#_ENREF_42), [47](#_ENREF_47), [51](#_ENREF_51)]  Mixed-methods  [[40](#_ENREF_40), [41](#_ENREF_41), [48](#_ENREF_48), [50](#_ENREF_50), [52](#_ENREF_52)] |
|  |  | Comorbidities | Living with another comorbidity (Diabetes, Glaucoma, CVD); Influence of PA on the management of comorbidities | **Emotion** (fear) | Comorbidities were experienced as both a barrier and facilitator. Improving the management of a comorbidity through PA was considered a facilitator for maintaining PA behaviour however, fear of exacerbating a comorbidity was considered a barrier.  "*Having a number of health issues that need exercise. If I don’t do it* [Exercise]*, I will regret it*" [[41](#_ENREF_41)]  "*The only thing is tiredness, because with the diabetes, at times, you can – it does – I think – it’s just a side effect of it that, you know, it’s – because you can’t – where anybody else can think, ‘I can have a...’ you can have an energy drink, I can’t*"[[27](#_ENREF_27)] | Qualitative  [[27](#_ENREF_27), [43](#_ENREF_43)]  Quantitative  [[51](#_ENREF_51)]  Mixed-methods  [[41](#_ENREF_41)] |
|  |  | Fear of injury | Fear of causing injury; fear of injury encouraging PA behaviour | **Emotion** (fear) | Fear of injury was considered both a barrier and a facilitator to maintaining PA. Individuals often feared that certain types of exercises may result in injury however some associated other types of exercise such as strength or stretching exercise for reducing injury risk.  “*As I say, on the odd occasion, coming down the stairs, it just gives me a bit of a jolt […] I do more of me stretches then and think ‘bloody hell, you know, I don’t want that coming back’. So instead of doing them perhaps once or twice a week I’ll do them every day for a week or a fortnight type of thing, you know what I mean?*” [[27](#_ENREF_27)]  “*Probably because I didn’t do the exercises. I did some exercises with trepidation for fear of causing my back pain…There was times that I just didn’t do the exercises due to other factors, whatever, at the time*” [[43](#_ENREF_43)] | Qualitative  [[27](#_ENREF_27), [43](#_ENREF_43)]  Quantitative  [[44](#_ENREF_44)] |
|  | Knowledge and beliefs | Knowledge of condition | Knowledge of condition; knowledge of impact of PA on condition | **Knowledge** (Knowledge of condition) | Knowledge of condition was considered both a barrier and facilitator. Some individuals with OA following a positive perception of their condition chose to no longer participate in PA as a result of an improved condition however this was a facilitator for those who deemed PA behaviour as helpful for managing their condition.  "*I must admit, towards the end, I did flag off a little bit. Mainly because my knee was feeling so good*” [[43](#_ENREF_43)]  "*I wanted to get rid of the pain. If the pain disappears, why would I bother to continue the exercises? I understand it is better to do the exercises to avoid the pain returning, but, if the pain returns, I will start the exercises again*” [[53](#_ENREF_53)]  "*I mean, whatever’s wrong in there is still wrong in there, but this is, this is enabling me to carry on with it, rather than giving into it, as it were. Does that make sense? […] and it’s meant I can keep my lifestyle, you know, it hasn’t stopped me doing anything*” [[27](#_ENREF_27)] | Qualitative  [[27](#_ENREF_27), [43](#_ENREF_43), [53](#_ENREF_53)]  Quantitative  [[42](#_ENREF_42)]  Mixed-methods  [[50](#_ENREF_50), [52](#_ENREF_52)] |
|  |  | Beliefs about pain | Beliefs about pain (non-physical); Knowledge of PA for pain. | **Beliefs about consequences** (characteristics of outcome expectancies) | Beliefs about pain were considered as a both barrier and facilitator to maintaining PA. This was considered a barrier for those associating PA with pain of their condition however PA behaviour that improved the management of pain, was considered a facilitator.  "*I probably learnt then that exercise is the way – you’ve got to work through the pain […] In the old days they used to say if you had a bad back, don’t exercise it, rest and I kind of, probably, maybe I’d got a bit of that thought about it. But now, I do exercise even on the days when I think, ‘Oh God, I’m dead, I don’t want to do it* [exercise] *today”[*[*27*](#_ENREF_27)*]*  “*Well, now I understand it is very important, understanding that a little bit of pain is OK and how to deal and manage that pain and understand that some pain to do with any sort of physical activity is OK and I’m not doing any further damage…*” [[43](#_ENREF_43)] | Qualitative  [[27](#_ENREF_27), [43](#_ENREF_43)]  Quantitative  [[47](#_ENREF_47)]  Mixed-methods  [[52](#_ENREF_52)] |
|  |  | Beliefs about consequences | Beliefs about consequences; feelings that PA increased other symptoms / conditions | **Beliefs about consequences** (consequents) | Beliefs about consequences was considered both a barrier and a facilitator to maintaining PA. This was considered a barrier for those associating negative affects following PA participation however a facilitator for those that consider PA as way to reduce / delay the consequences they associate with OA.  "*I thought it was doing me OK but then no, I just couldn’t deal with it anymore…I ended up having to have injections in my hips afterwards. Because I do have bursitis in my hips, so it actually created more problems for me*” [[43](#_ENREF_43)]  "*I mean, you can tell with your body, as you get older, that it’s not the same as it was and you stiffen up and everything. A lot more things are a lot, are painful to do, but, I just – if I can possibly avoid it, I don’t want to get any worse*” [[27](#_ENREF_27)]  "*It’s made me resolve to forego any knee surgery if I possibly can and certainly not allow an increase in weight to put me back in a greater risk of knee surgery*" [[45](#_ENREF_45)] | Qualitative  [[27](#_ENREF_27), [43](#_ENREF_43), [45](#_ENREF_45)]  Mixed-methods  [[48](#_ENREF_48)] |
|  | Psychological factors | Self-efficacy | High self-efficacy such as belief in successful execution of PA; low self-efficacy such as lack of belief in PA execution. | **Beliefs about capabilities** (self-efficacy) | Self-efficacy was reported as both a facilitator and a barrier to maintaining PA behaviour. This was seen as a facilitator for those that showed high levels of self-efficacy through belief in successful execution of PA; however, it was considered a barrier in those that exhibited low levels of self-efficacy or low belief in successful PA execution.  "*Probably because my performance is nothing to write home about. It can damn well be that if I had been able to lift loads of 5, 10 kg or more than the others, then it would have been more fun…*” [[50](#_ENREF_50)]  “*On my own is very lazy, say I will do but never do…” [*[*48*](#_ENREF_48)*]*  “*But we covered the book and it got me to a stage where I was comfortable doing my exercise and there was nothing of concern really…*” [[43](#_ENREF_43)]  “*It got me where I had a wide variety of different exercises that I could do and I felt supported and I knew what I needed to do, so I didn’t really need more* [physio consults]” [[43](#_ENREF_43)] | Qualitative  [[27](#_ENREF_27), [43](#_ENREF_43), [45](#_ENREF_45), [53](#_ENREF_53)]  Quantitative  [[42](#_ENREF_42), [47](#_ENREF_47), [49](#_ENREF_49)]  Mixed-methods  [[40](#_ENREF_40), [41](#_ENREF_41), [48](#_ENREF_48), [50](#_ENREF_50), [52](#_ENREF_52)] |
|  |  | Motivation | Low or high motivation to maintain PA behaviour; motivation reinforced through goals and/or perceived benefits | **Reinforcement** (Reinforcement) | Motivation was considered both a barrier and a facilitator to maintaining PA behaviour. In individuals where motivation was high and they were reaching achievable goals; this reinforced motivation to continue with PA behaviour. In individuals where motivation was low or whereby motivation decreased due to not feeling capable / able to reach their goals, this impacted their ability to maintain PA behaviour. Furthermore, motivation was also considered as part of a feedback loop as motivation to participate in PA often led to increased confidence in performing PA and in reaching goals which reinforced the motivation to maintain PA behaviour.  "*After the program finished you know, it wasn’t hard to maintain it, and I still do a lot of walking and you know I’ve been to some strength training and stuff like that and actually I’m doing running and that was my aim, to be able to run*" [[45](#_ENREF_45)]  "*I continue with my exercise…. I really know these exercises have beneficial effects and that motivates me to continue with my exercises. The main motivation to do all this is to prevent an operation to get a new hip*" [[53](#_ENREF_53)]  "*Because it was a knee pain thing that I’m doing, I thought of the weight loss as a bonus. I was concentrating on getting the leg right—It would give me an opportunity to try an alternative to surgery, so I kept on track for that…Then eventually the knee was getting really good and the weight loss was happening so it kept me motivated to keep on going*"[[45](#_ENREF_45)] | Qualitative  [[27](#_ENREF_27), [43](#_ENREF_43), [45](#_ENREF_45), [46](#_ENREF_46), [53](#_ENREF_53)]  Quantitative  [[42](#_ENREF_42), [47](#_ENREF_47)]  Mixed-methods  [[40](#_ENREF_40), [41](#_ENREF_41), [50](#_ENREF_50), [52](#_ENREF_52)] |
|  |  | Perceived benefits | Improved ability in performing activities; reducing need for surgery; weight-loss; reducing medication use | **Beliefs about capabilities** (perceived behavioural control) | Perceived benefits were considered a facilitator for maintaining PA behaviour. Individuals often associated the benefits they experienced from PA with helping them in the management of their condition, their ability to perform everyday activities, reducing medication needs and potential for reducing surgical intervention. This perceived behavioural control to be able to manage and improve their condition was considered a facilitator to maintaining PA behaviour.  "*I can go without medication if I do stretching. [I can] relax and forget about everything that is going on*" [[41](#_ENREF_41)]  “*I thought that it would be a good thing if I could strengthen some muscles. That would make things easier and maybe delay the need for surgery and so on. Now I’m 100% certain about that*" [[50](#_ENREF_50)] | Qualitative  [[45](#_ENREF_45), [46](#_ENREF_46), [53](#_ENREF_53)]  Quantitative  [[42](#_ENREF_42)]  Mixed-methods  [[41](#_ENREF_41), [50](#_ENREF_50)] |
|  |  | Active Identity | Association of PA as part of their identity; PA perceived as normative behaviour; Assuming responsibility of PA to manage OA | **Social / professional role and identity** (identity) | Active identity was considered a facilitator to maintaining PA behaviour within this population. Those who described themselves as being an active person was perceived as a facilitator to maintaining PA behaviour. Topics such as describing feelings of guilt (internalising responsibility for action) when they did not perform the whole programme and assuming responsibility to self-manage OA through PA were seen as facilitators to maintaining PA behaviour. Furthermore, this was also seen as a feedback loop as those that exhibited PA behaviour as part of their identity often positioned themselves as the primary managers of their condition, reinforcing their PA participation to manage their OA.  “*I feel very guilty if I don’t do it with my full program*” [[41](#_ENREF_41)]  "*It’s actually quite hard to separate the mind from the body when you exercise because you have such great conscience after you have exercised…*” [[50](#_ENREF_50)]  “*The one thing that I’ve learnt is, from the study is, that the more exercise I do, the more I do with it, I think the better it’s gonna be, rather than nursing it, keep doing it and keep exercising it and so that’s what I do*” [[27](#_ENREF_27)]  “*I wouldn’t trouble him [GP], because I know the remedy’s in my own hands, in terms of the maintenance exercise regime*” [[27](#_ENREF_27)] | Qualitative  [[27](#_ENREF_27), [45](#_ENREF_45), [53](#_ENREF_53)]  Mixed-methods  [[41](#_ENREF_41), [50](#_ENREF_50)] |
|  | Behavioural Outcomes | Coping strategies | Developing actions to overcome barriers to PA | **Behavioural regulation** (action planning) | Coping strategies was seen as a facilitator for maintaining PA behaviour for this population. This was similar to integration into daily life as it evolved around developing action for routine formation however differed in that coping strategies described methods implemented to overcome challenges / barriers that may disrupt routine or habit formation such as exercising at work or throughout the day when routine habit formation may not be considered feasible.  "*I combined yoga with physical therapy . . . I do most of my yoga in bed before I get up because it is hard to get down on the floor"*" [[41](#_ENREF_41)]  "*…If you didn’t have time to do all of ‘em, you could just start some of them during the day, just as long as you finished it during the day, and I know I would get tired, and I said, ‘Oh no. I forgot to do the lunges,’ you know, that was the last thing, OK. But you know, if I started out in the morning* [leaving her home]*, I was doing them everywhere… I would go quilting, and… I see the stairway* [to perform step-up exercises]" [[46](#_ENREF_46)] | Qualitative  [[27](#_ENREF_27), [43](#_ENREF_43), [46](#_ENREF_46)]  Mixed-methods  [[41](#_ENREF_41)] |
|  |  | Development of exercise skills | Capabilities and skills developed in performing exercises; moderating to exercises that were more achievable | **Skills** (skill development) | Development of exercise skills was considered both a barrier and facilitator to maintaining PA behaviour. For individuals that found certain PA behaviours difficult, this was considered a barrier; however, for individuals that described skill acquisition from PA, this reinforced their confidence and ability to perform PA behaviour, acting as a facilitator and facilitated a feedback loop of skill development, reinforcing confidence in PA performance.  “*But we covered the book* [Exercise book] *and it got me to a stage where I was comfortable doing my exercise and there was nothing of concern really…*” [[43](#_ENREF_43)]  “*I was able to negotiate away from the ones that were awkward or difficult for me, to the ones that were easier or more present or physically possible in my house*” [[43](#_ENREF_43)]  "*So the fact that I know I can do it is quite a motivator now…I think I now have the confidence and knowledge and understanding to be able to improve my health again. Even though at the moment I’m not achieving that how I would like to, I am confident that I can. I think that’s a significant part of the programme*" [[45](#_ENREF_45)] | Qualitative  [[27](#_ENREF_27), [43](#_ENREF_43), [45](#_ENREF_45), [53](#_ENREF_53)]  Quantitative  [[42](#_ENREF_42)]  Mixed-methods  [[52](#_ENREF_52)] |
|  |  | Empowerment and Autonomy | Autonomy; empowering individuals to independently complete ADLs; growing self-confidence | **Beliefs about capabilities** (empowerment) | Empowerment and autonomy were described as facilitators. Individuals with hip and knee OA were empowered by their capabilities and in understanding how to self-manage their condition. This informed us that developing autonomy empowers individuals to continue participation with PA behaviour.  “*Yoga prepares my body to do more. I recently joined a curling club, something I have always wanted to do . . . don’t think I would be able to do it if I had not done yoga*” [[40](#_ENREF_40)]  "*When I exercise it certainly gets stronger, and I think with the strength, also, I think I’ve grown more confident on my knee overall*” [[27](#_ENREF_27)] | Qualitative  [[27](#_ENREF_27), [43](#_ENREF_43)]  Quantitative  [[47](#_ENREF_47)]  Mixed-methods  [[40](#_ENREF_40), [48](#_ENREF_48), [52](#_ENREF_52)] |
|  |  | Improved function and Q.O. L | Improvement in function; quality of life; performing work related activities | **Behavioural regulation** (self-monitoring) | Improved function and quality of life was considered a facilitator. Individuals often associated PA as encouraging as it helped them to self-manage their condition and to be able to do more activities such as housework, work, caring responsibilities and engage in other activities such as travel.  “*I felt so much stronger. I could barely walk, I’d use a walker – inside the house, just to go to the sink. And when I first went there I could barely walk and I was doing just a few hundred steps a day…And then I worked up to 6,000 and – I could walk to the sink without my walker. So I definitely got improvement as far as strength went.*” [[43](#_ENREF_43)]  “*I just got back from traveling to Europe with my granddaughter and was nervous about all the walking, but I didn’t have that much pain in my knees and was able to keep up with her and enjoy the trip*” [[40](#_ENREF_40)]. | Qualitative  [[27](#_ENREF_27), [43](#_ENREF_43)]  Quantitative  [[47](#_ENREF_47)]  Mixed-methods  [[40](#_ENREF_40), [41](#_ENREF_41), [52](#_ENREF_52)] |
|  |  | Integration into daily life | Habit formation; scheduling PA into daily activities; facilitating habit formation | **Behavioural regulation** (self-monitoring) | Integrating PA behaviour into daily life was seen as a facilitator to maintaining PA behaviour. In individuals with hip and/or knee OA, the integration of PA behaviour into daily life and activities was often performed through routine habit formation such as exercising before bed, every night or routine scheduled PA participation.  "*I am very much of a habit person; I have exercise scheduled into my life*" [[41](#_ENREF_41)].  “*I felt, as I worked on the exercises – like religiously, doing them every second day*” [[43](#_ENREF_43)]  "*It was an easy process for me, because I had everything laid out. I kept my bag right there in the kitchen, so while I’m in the kitchen, before I start my day, I would just do my exercises*" [[46](#_ENREF_46)] | Qualitative  [[43](#_ENREF_43), [46](#_ENREF_46)]  Mixed-methods  [[41](#_ENREF_41), [48](#_ENREF_48), [52](#_ENREF_52)] |
| Social Factors | Support systems | Family, peers, Physiotherapists | Peer support; physiotherapist follow-up; therapeutic alliance; family encouragement | **Social influences** (social support) | Support systems in the form of family, peers and physiotherapists was considered both a facilitator and a barrier to maintaining PA behaviour. Individuals often described family members reminding them to complete their exercise or physiotherapists providing additional support in the form of follow-up. Conversely, a lack of trust in physiotherapist and little support acted as a barrier.  "*My wife. She’d say sometimes, ‘I haven’t seen you do your exercises.’ And it may be that I’d done them when she wasn’t around, and I’d say, ‘Oh, yeah, I did them this morning,’ or, ‘I did them,’ you know, whatever. And then I’d go, another time I’d go [tuts], ‘Yeah, fair cop, guv,’ you know and off we go. So, having someone to know what the regime is, I suppose if you’ve got good communications and, you know. So that helped, having another person in the house saying, ‘I haven’t seen you do them,’ which is code for, ‘Go and do them’*’ [[27](#_ENREF_27)]  "*My daughter told me, ‘Mom you need to do yoga, you need to do yoga’* ” [[41](#_ENREF_41)] | Qualitative  [[27](#_ENREF_27), [45](#_ENREF_45), [46](#_ENREF_46)]  Mixed-methods  [[41](#_ENREF_41), [50](#_ENREF_50), [52](#_ENREF_52)] |
|  |  | Accountability | Feelings of accountability to self, and other; External accountability such as self-monitoring with pedometers | **Social influences** (social support) | Accountability was considered a facilitator to maintaining PA behaviour. Individuals with OA often described the impact of their therapist on influencing their PA and through the accountability of others as part of exercise groups.  "*Just having that consultation and someone working with you along the way, there’s a sense of obligation to yourself and to the other person*” [[43](#_ENREF_43)]  "*The thing is you do the exercise ‘cause you feel that you don’t want to let the other person down. You know you do them ‘cause in the first instance you think, ‘Oh that’s going to do me good, it’s going to yeah’, but also there’s a secondary thing there you think, ‘Oh he’s gone out of his way to explain these things to me and shown me what to do it’s only fair that I do them so at least I can tell him what sort of effect its having the next time I meet him’, you know*" [[27](#_ENREF_27)] | Qualitative  [[27](#_ENREF_27), [43](#_ENREF_43), [45](#_ENREF_45), [46](#_ENREF_46)]  Mixed-methods  [[41](#_ENREF_41), [52](#_ENREF_52)] |
|  |  | Group identity | Associations with being part of a group; group identity reinforcing accountability | **Social influences** (group identity) | Group identity was considered a facilitator to maintaining PA behaviour. PA in the form of exercising as part of a group was described to positively impact PA behaviour. This encouraged individuals to participate in PA. This was also seen as a feedback loop whereby identifying with the group reinforced motivation for each other to perform PA behaviour.  “*There are other people around. We sort of inspire one another*" [[41](#_ENREF_41)]  "*So in general you keep in mind that you were involved in that trial and that it was nice to have that opportunity. It’s a bit like being back at school, where you don’t have to pay school fees, and so you feel obliged to also get something out of it in one way or another*" [[43](#_ENREF_43)] | Qualitative  [[43](#_ENREF_43)]  Mixed-methods  [[41](#_ENREF_41), [52](#_ENREF_52)] |
|  |  | Social isolation | Social isolation in the form of: lack of exercise partners; missing group-based activities | **Social influences** (alienation) | Lack of an exercise partner or lack of encouragement and/or follow-up after a programme often led to feelings of social isolation which was perceived as a barrier to maintaining PA behaviour for the hip and knee OA population.  "*Because it makes a difference. It’s more motivating when you’re doing it [exercise] with someone else. It’s easy to go back to your regular routine of doing nothing. I mean, if it’s just me, I don’t, I don’t care about me. [laughs] But it’s different, I’d care about someone else*" [[46](#_ENREF_46)]  "*I’m not real good if I’m not accountable to somebody or something…Once I had no contact with anybody I felt like I’d been left alone and nobody cared what I did so I didn’t care what I did if you know what I mean…*” [[45](#_ENREF_45)]  "*I missed the group. Maybe sometimes we could have, during the interim, have one group study again, come in and see how everybody’s doing, and then go back…*” [[46](#_ENREF_46)] | Qualitative  [[27](#_ENREF_27), [45](#_ENREF_45), [46](#_ENREF_46), [53](#_ENREF_53)]  Quantitative  [[42](#_ENREF_42)]  Mixed-methods  [[50](#_ENREF_50), [52](#_ENREF_52)] |
| Environmental Factors | Access and Resources | Time constraints | Lack of time; other responsibilities taking up time; feelings of being too busy to perform PA | **Environmental Context and resources** (barriers) | Time constraints due to other commitments such as caring responsibilities, changes in lifestyle and work often acted as a barrier to maintaining PA behaviour for individuals with hip and knee OA.  "*I don’t do a lot of yoga because of time restraints*" [[41](#_ENREF_41)]  “*I’m too busy. I don’t want to spend the time . .* *.*" [[41](#_ENREF_41)]  "*Probably just time and work and you just sort of forget you go ‘oh I’ll do it later’ and then later comes and you forget don’t you?*" [[27](#_ENREF_27)] | Qualitative  [[27](#_ENREF_27), [43](#_ENREF_43)]  Quantitative  [[39](#_ENREF_39), [42](#_ENREF_42), [44](#_ENREF_44)]  Mixed-methods  [[40](#_ENREF_40), [41](#_ENREF_41), [48](#_ENREF_48), [52](#_ENREF_52)] |
|  |  | Cost and facilities available | Lack of access / available resources; cost and travel related barriers | **Environmental Context and resources** (barriers) | Lack of access to PA opportunities and resources were described as barriers to maintaining PA behaviour. Conversely, having access to these services such as tapping into local exercise classes and the availability of exercise machines were considered facilitators to maintaining PA behaviour.  "*There is a need for some mobile, verbal things you can just tap into instantaneously*" [[41](#_ENREF_41)]  "*I go to Curves, I try to go two or three times a week, and if I don’t do that, I do the other exercises . . . I also bowl like two or three times a week*" [[41](#_ENREF_41)] | Quantitative  [[42](#_ENREF_42), [47](#_ENREF_47), [49](#_ENREF_49)]  Mixed-methods  [[41](#_ENREF_41), [52](#_ENREF_52)] |
|  |  | Resources from intervention | Resources available following initial intervention such as exercise programmes, website resources and devices | **Environmental Context and resources** (Resources/material resources) | Resources from the intervention were described as largely positive and considered a facilitator for encouraging individuals to maintain PA behaviour. These appeared in the form of exercise sheets / books, automated text-message support, tape recordings and activity trackers  “*This is an activity that I actually can do. I use those home practice instruction sheet[s] we got for the study*” [[41](#_ENREF_41)]  "*If you had any problem, you had a take home manual, if you go through that, that would help you…if you forgot the procedure, you know, the right way to stand – or whatever –*” [[46](#_ENREF_46)]  "*It [BOOST-TLC] would ask you more specific questions, how many times did you exercise, what were your goals, and I thought that was good. It was kind of a pain in the neck sometimes [laughs]. I said “Oh, I don’t feel like doing this,” but I thought it was good, because it made me think, “OK. When am I gonna exercise?*" [[46](#_ENREF_46)] | Qualitative  [[43](#_ENREF_43), [45](#_ENREF_45), [46](#_ENREF_46)]  Mixed-methods  [[41](#_ENREF_41), [48](#_ENREF_48)] |
|  | Unexpected events | Salient / critical events | Unexpected events such as surgery; immediate caring responsibilities; lifestyle changes; fluctuations in health that are non-modifiable | **Environmental Context and resources** (salient / critical events) | Salient or critical events whereby individuals experienced major health fluctuations, unexpected caring responsibilities or unexpected accidents acted as a barrier to maintaining PA.  "*I had glaucoma . . . for a few months I was unable to do yoga . .* ." [[41](#_ENREF_41)]  "*My disabled daughter continues to [need]a great deal . . . my husband got prostate cancer . . .*" [[41](#_ENREF_41)]  "*I had to have a hip replacement. So, for the last six months I have not been able to do terribly much…*” [[45](#_ENREF_45)] | Qualitative  [[43](#_ENREF_43), [45](#_ENREF_45), [46](#_ENREF_46)]  Mixed-methods  [[40](#_ENREF_40), [41](#_ENREF_41)] |
|  |  | Weather | Poor weather conditions | **Environmental Context and resources** (barriers) | Poor seasonal weather conditions such as winter often limited participants motivation to participate in PA behaviour.  "*It’s so easy to, to vegetate in front of the telly, isn’t it? You’ve gotta really – particularly in the winter months – you’ve gotta really force yourself to get out there and, and do something [hmm], rather than just shutting yourself away*” [[27](#_ENREF_27)] | Qualitative  [[27](#_ENREF_27)]  Quantitative  [[42](#_ENREF_42)] |

## **Quality Appraisal –** Mixed Methods Appraisal Tool (MMAT)

**Supplementary table 6. MMAT - Quality Appraisal - Qualitative studies**

| 1. Qualitative studies (qualitative studies) – Mixed Methods Appraisal Tool (MMAT), [Yes, no, can’t tell for each question] | | | | | | | |
| --- | --- | --- | --- | --- | --- | --- | --- |
| Study (First author, year) | S1. Are there clear research questions? | S2. Do the collected data allow to address the research questions? | 1.1. Is the qualitative approach appropriate to answer the research question? | 1.2. Are the qualitative data collection methods adequate to address the research question? | 1.3. Are the findings adequately derived from the data? | 1.4. Is the interpretation of results sufficiently substantiated by data? | 1.5. Is there coherence between qualitative data sources, collection, analysis and interpretation? |
| Cheung, 2022 | Yes | Yes | Yes | Yes | Yes | Yes | Yes |
| Cheung, 2015 | Yes | Yes | Yes | Yes | Can't tell | Yes | Can't tell |
| Hammer, 2015 | Yes | Yes | Yes | Yes | Yes | Yes | Yes |
| Hinman, 2023 | Yes | Yes | Yes | Yes | Yes | Yes | Yes |
| Lawford, 2023 | Yes | Yes | Yes | Yes | Yes | Yes | Yes |
| Ledingham, 2020 | Yes | Yes | Yes | Yes | Yes | Yes | Yes |
| Matile, 2024 | Yes | Yes | Yes | Yes | Yes | Can't tell | Can't tell |
| Moore, 2020 | Yes | Yes | Yes | Yes | Yes | Yes | Yes |
| Veenhof, 2006 | Yes | Yes | Yes | Yes | Yes | Yes | Yes |
| Tan, 2025 | Yes | Yes | Yes | Yes | Yes | Yes | Yes |

**Supplementary table 7. MMAT** **- Quality Appraisal - Quantitative - Randomised controlled trials**

| 2. Quantitative studies (Randomised controlled trials) – Mixed Methods Appraisal Tool (MMAT), [Yes, no, can’t tell for each question] | | | | | | | |
| --- | --- | --- | --- | --- | --- | --- | --- |
| Study (First author, Year) | S1. Are there clear research questions? | S2. Do the collected data allow to address the research questions? | 2.1. Is randomization appropriately performed? | 2.2. Are the groups comparable at baseline? | 2.3. Are there complete outcome data? | 2.4. Are outcome assessors blinded to the intervention provided? | 2.5 Did the participants adhere to the assigned intervention? |
| Bennell, 2020 | Yes | Yes | Yes | Yes | Yes | Yes | Yes |
| Hinman, 2020 | Yes | Yes | Yes | Yes | Yes | Yes | Yes |
| Stanton, 2020 | Yes | Yes | Yes | No | No | Yes | Yes |

**Supplementary table 8. MMAT - Quality Appraisal - Quantitative - Non-randomised trials**

| 3. Quantitative studies (Non randomised trials) – Mixed Methods Appraisal Tool (MMAT), [Yes, no, can’t tell for each question] | | | | | | | |
| --- | --- | --- | --- | --- | --- | --- | --- |
| Study (First author, Year) | S1. Are there clear research questions? | S2. Do the collected data allow to address the research questions? | 3.1. Are the participants representative of the target population? | 3.2. Are measurements appropriate regarding both the outcome and intervention (or exposure)? | 3.3. Are there complete outcome data? | 3.4. Are the confounders accounted for in the design and analysis? | 3.5. During the study period, is the intervention administered (or exposure occurred) as intended? |
| Cheung, 2022 | Yes | Yes | Can't tell | Yes | Can't tell | No | Yes |
| Cheung, 2015 | Yes | Yes | Can't tell | Yes | Yes | No | Yes |
| Desai, 2014 | Yes | Yes | Can't tell | Yes | No | Yes | Yes |
| Hammer, 2015 | Yes | Yes | Can't tell | Yes | Yes | No | Yes |
| Kawi, 2015 | Yes | Yes | Yes | Yes | Yes | Can't tell | Yes |
| Matile, 2024 | Yes | Yes | Yes | Can't tell | Yes | Yes | Yes |

**Supplementary table 9. MMAT - Quality Appraisal - Quantitative - Descriptive design studies**

| 4. Quantitative studies (Quantitative descriptive studies) – Mixed Methods Appraisal Tool (MMAT), [Yes, no, can’t tell for each question] | | | | | | | |
| --- | --- | --- | --- | --- | --- | --- | --- |
| Study (First author, Year) | S1. Are there clear research questions? | S2. Do the collected data allow to address the research questions? | 4.1. Is the sampling strategy relevant to address the research question? | 4.2. Is the sample representative of the target population? | 4.3. Are the measurements appropriate? | 4.4. Is the risk of nonresponse bias low? | 4.5. Is the statistical analysis appropriate to answer the research question? |
| Wainwright, 2020 | Yes | Yes | Yes | Yes | No | Can’t tell | Can’t tell |
| Tan, 2025 | Yes | Yes | Yes | Yes | Yes | Can’t tell | Yes |

**Supplementary table 10. MMAT - Quality Appraisal - Mixed-methods studies**

| 5. Mixed-methods studies (Mixed-methods components) – Mixed Methods Appraisal Tool (MMAT), [Yes, no, can’t tell for each question] | | | | | | | |
| --- | --- | --- | --- | --- | --- | --- | --- |
| Study (First author, Year) | S1. Are there clear research questions? | S2. Do the collected data allow to address the research questions? | 5.1. Is there an adequate rationale for using a mixed methods design to address the research question? | 5.2. Are the different components of the study effectively integrated to answer the research question? | 5.3. Are the outputs of the integration of qualitative and quantitative components adequately interpreted? | 5.4. Are divergences and inconsistencies between quantitative and qualitative results adequately addressed? | 5.5. Do the different components of the study adhere to the quality criteria of each tradition of the methods involved? |
| Cheung, 2022 | Yes | Yes | Yes | Yes | Yes | No | Yes |
| Cheung, 2015 | Yes | Yes | No | No | Yes | Yes | Yes |
| Hammer, 2015 | Yes | Yes | Yes | Yes | Yes | Yes | Yes |
| Matile, 2024 | Yes | Yes | Yes | Yes | Yes | Yes | Can't tell |
| Tan, 2025 | Yes | Yes | Can’t tell | Yes | Yes | Yes | Yes |

**Supplementary figure 1. Quality assessment using the Mixed-methods Appraisal Tool (MMAT)**

## **PRISMA Checklist**

| **Section and Topic** | **Item #** | **Checklist item** | **Location where item is reported** |
| --- | --- | --- | --- |
| **TITLE** | | |  |
| Title | 1 | Identify the report as a systematic review. | Title page |
| **ABSTRACT** | | |  |
| Abstract | 2 | See the PRISMA 2020 for Abstracts checklist. | Page 1-2, Abstract |
| **INTRODUCTION** | | |  |
| Rationale | 3 | Describe the rationale for the review in the context of existing knowledge. | Page 2-4, introduction |
| Objectives | 4 | Provide an explicit statement of the objective(s) or question(s) the review addresses. | Page 4, introduction |
| **METHODS** | | |  |
| Eligibility criteria | 5 | Specify the inclusion and exclusion criteria for the review and how studies were grouped for the syntheses. | Page 5, methods |
| Information sources | 6 | Specify all databases, registers, websites, organisations, reference lists and other sources searched or consulted to identify studies. Specify the date when each source was last searched or consulted. | Page 4-5, methods |
| Search strategy | 7 | Present the full search strategies for all databases, registers and websites, including any filters and limits used. | Supplementary material |
| Selection process | 8 | Specify the methods used to decide whether a study met the inclusion criteria of the review, including how many reviewers screened each record and each report retrieved, whether they worked independently, and if applicable, details of automation tools used in the process. | Page 5-6, methods |
| Data collection process | 9 | Specify the methods used to collect data from reports, including how many reviewers collected data from each report, whether they worked independently, any processes for obtaining or confirming data from study investigators, and if applicable, details of automation tools used in the process. | Page 5-6, methods |
| Data items | 10a | List and define all outcomes for which data were sought. Specify whether all results that were compatible with each outcome domain in each study were sought (e.g. for all measures, time points, analyses), and if not, the methods used to decide which results to collect. | Page 6, methods |
|  | 10b | List and define all other variables for which data were sought (e.g. participant and intervention characteristics, funding sources). Describe any assumptions made about any missing or unclear information. | Page 6, methods |
| Study risk of bias assessment | 11 | Specify the methods used to assess risk of bias in the included studies, including details of the tool(s) used, how many reviewers assessed each study and whether they worked independently, and if applicable, details of automation tools used in the process. | Page 6-7, methods; page 13, results |
| Effect measures | 12 | Specify for each outcome the effect measure(s) (e.g. risk ratio, mean difference) used in the synthesis or presentation of results. | Page 7, methods; page 9, results |
| Synthesis methods | 13a | Describe the processes used to decide which studies were eligible for each synthesis (e.g. tabulating the study intervention characteristics and comparing against the planned groups for each synthesis (item #5)). | Page 5-7, methods |
|  | 13b | Describe any methods required to prepare the data for presentation or synthesis, such as handling of missing summary statistics, or data conversions. | Page 6-7, methods |
|  | 13c | Describe any methods used to tabulate or visually display results of individual studies and syntheses. | Page 6-7, methods |
|  | 13d | Describe any methods used to synthesize results and provide a rationale for the choice(s). If meta-analysis was performed, describe the model(s), method(s) to identify the presence and extent of statistical heterogeneity, and software package(s) used. | Pages 6-7, methods |
|  | 13e | Describe any methods used to explore possible causes of heterogeneity among study results (e.g. subgroup analysis, meta-regression). | Page 6-7, methods |
|  | 13f | Describe any sensitivity analyses conducted to assess robustness of the synthesized results. | N/A |
| Reporting bias assessment | 14 | Describe any methods used to assess risk of bias due to missing results in a synthesis (arising from reporting biases). | N/A |
| Certainty assessment | 15 | Describe any methods used to assess certainty (or confidence) in the body of evidence for an outcome. | N/A |
| **RESULTS** | | |  |
| Study selection | 16a | Describe the results of the search and selection process, from the number of records identified in the search to the number of studies included in the review, ideally using a flow diagram. | figure 1 - flow chart; page 9, results |
|  | 16b | Cite studies that might appear to meet the inclusion criteria, but which were excluded, and explain why they were excluded. | Reasons for exclusion on Page 8, figure 1 |
| Study characteristics | 17 | Cite each included study and present its characteristics. | Page 10-11, table 1; Supplementary material. |
| Risk of bias in studies | 18 | Present assessments of risk of bias for each included study. | Page 9, results; Page 10-11, table 1; supplementary material |
| Results of individual studies | 19 | For all outcomes, present, for each study: (a) summary statistics for each group (where appropriate) and (b) an effect estimate and its precision (e.g. confidence/credible interval), ideally using structured tables or plots. | Pages 10-11, table 1; and table 2, figure 2, table 3 |
| Results of syntheses | 20a | For each synthesis, briefly summarise the characteristics and risk of bias among contributing studies. | Page 9-11, table 1; page 12-16, results |
|  | 20b | Present results of all statistical syntheses conducted. If meta-analysis was done, present for each the summary estimate and its precision (e.g. confidence/credible interval) and measures of statistical heterogeneity. If comparing groups, describe the direction of the effect. | N/A |
|  | 20c | Present results of all investigations of possible causes of heterogeneity among study results. | Pages 12-24, table 1; table 2, figure 2, table 3 |
|  | 20d | Present results of all sensitivity analyses conducted to assess the robustness of the synthesized results. | N/A |
| Reporting biases | 21 | Present assessments of risk of bias due to missing results (arising from reporting biases) for each synthesis assessed. | N/A |
| Certainty of evidence | 22 | Present assessments of certainty (or confidence) in the body of evidence for each outcome assessed. | N/A |
| **DISCUSSION** | | |  |
| Discussion | 23a | Provide a general interpretation of the results in the context of other evidence. | Discussion, Page 25-27 |
|  | 23b | Discuss any limitations of the evidence included in the review. | Discussion, pages 27-28 |
|  | 23c | Discuss any limitations of the review processes used. | Discussion, pages 27-28 |
|  | 23d | Discuss implications of the results for practice, policy, and future research. | Discussion, pages 28-29 |
| **OTHER INFORMATION** | | |  |
| Registration and protocol | 24a | Provide registration information for the review, including register name and registration number, or state that the review was not registered. | Methods, page 4-5 |
|  | 24b | Indicate where the review protocol can be accessed, or state that a protocol was not prepared. | Methods, page 4-5 |
|  | 24c | Describe and explain any amendments to information provided at registration or in the protocol. | N/A |
| Support | 25 | Describe sources of financial or non-financial support for the review, and the role of the funders or sponsors in the review. | Statements and declarations, page 2 |
| Competing interests | 26 | Declare any competing interests of review authors. | Statements and declarations, page 2 |
| Availability of data, code and other materials | 27 | Report which of the following are publicly available and where they can be found: template data collection forms; data extracted from included studies; data used for all analyses; analytic code; any other materials used in the review. | Supplementary material |

*Adapted from:*  Page MJ, McKenzie JE, Bossuyt PM, Boutron I, Hoffmann TC, Mulrow CD, et al. The PRISMA 2020 statement: an updated guideline for reporting systematic reviews. BMJ 2021;372:n71. doi: 10.1136/bmj.n71. This work is licensed under CC BY 4.0. To view a copy of this license, visit <https://creativecommons.org/licenses/by/4.0/>

1. CHAIN, Cycling against Hip Pain; EARS, Exercise Adherence Rating Scale; GLA:D, Good living with Osteoarthritis from Denmark; KOOS, Knee osteoarthritis outcome score; MMAT, Mixed-methods appraisal tool; OA, Osteoarthritis; PA, Physical Activity; PPI, Participant and Patient Involvement; RCT, Randomised Control Trial; SMS, Short message service; TR, Telephone Reinforcement; UK, United Kingdom; USA, United States of America [↑](#footnote-ref-1)
